# Time-synchronic comments on video streaming website reveal core structures of audience engagement in movie viewing

**DOI:** 10.3389/fpsyg.2022.1040755

**Published:** 2023-01-19

**Authors:** Wenjing Ni, Christophe Coupé

**Affiliations:** ^1^Department of Linguistics, School of Humanities, The University of Hong Kong, Pokfulam, Hong Kong SAR, China; ^2^Laboratoire Dynamique du Langage, UMR 5596-CNRS, Université Lyon 2, Lyon, France

**Keywords:** social media, psychology, natural language processing, human media interaction, media psychology

## Abstract

To what extent movie viewers are swept into a fictional world has long been pondered by psychologists and filmmakers. With the development of time-synchronic comments on online viewing platforms, we can now analyze viewers’ immediate responses toward movies. In this study, we collected over 3 million Chinese time-synchronic comments from a video streaming website. We first assessed emotion and cognition-related word rates in these comments with the Simplified Chinese version of the Linguistic Inquiry and Word Count (SCLIWC) and applied time-series clustering to the word rates. Then Hierarchical Density-Based Spatial Clustering of Applications with Noise (HDBSCAN) was conducted on the text to investigate the prevalent topics among the comments. We found different commenting behaviors in front of various movies and prototypical diachronic trajectories of the psychological engagement of the audience. We further identified how topics are discussed through time, and tried to account for viewer’s engagement, considering successively movie genres, topics and movie content. Among other points, we finally discussed the challenge in explaining the trajectories of engagement and the disconnection with narrative content. Overall, our study provides a new perspective on using social media data to answer questions from psychology and film studies. It underscores the potential of time-synchronic comments as a resource for detecting real-time human responses to specific events.

## Introduction

Scholars in psychocinematics are curious about people’s cognition, brain activity, and emotions when viewing movies (Hasson et al., 2008; Oatley, 2013; Plantinga, 2013; Tan, 2018). It has been shown that movies can successfully elicit the audience’s amusement, anger, contentment, disgust, sadness, etc., under laboratory conditions (Philippot, 1993; Gross and Levenson, 1995; Gilman et al., 2017; Maffei and Angrilli, 2019). However, experimental studies focusing on physiological reactions and behaviors seldom investigate the content of viewers’ mental states. Text, on the other hand, is a more accessible resource to start with. For instance, viewers leave textual evidence of their thinking and feeling about films in the form of movie reviews. With Natural Language Processing (NLP) methods, scholars from linguistics, psychology, computer science, and arts have explored crowd opinion toward a specific film and how, for example, viewers like or dislike a character or a star (Chaovalit and Zhou, 2005; Zhuang et al., 2006; Lee et al., 2016; Topal and Ozsoyoglu, 2016). Nevertheless, despite the detailedness of traditional movie reviews, they only partially reflect viewers’ instant responses to specific time points in a movie.

**Figure 1:**
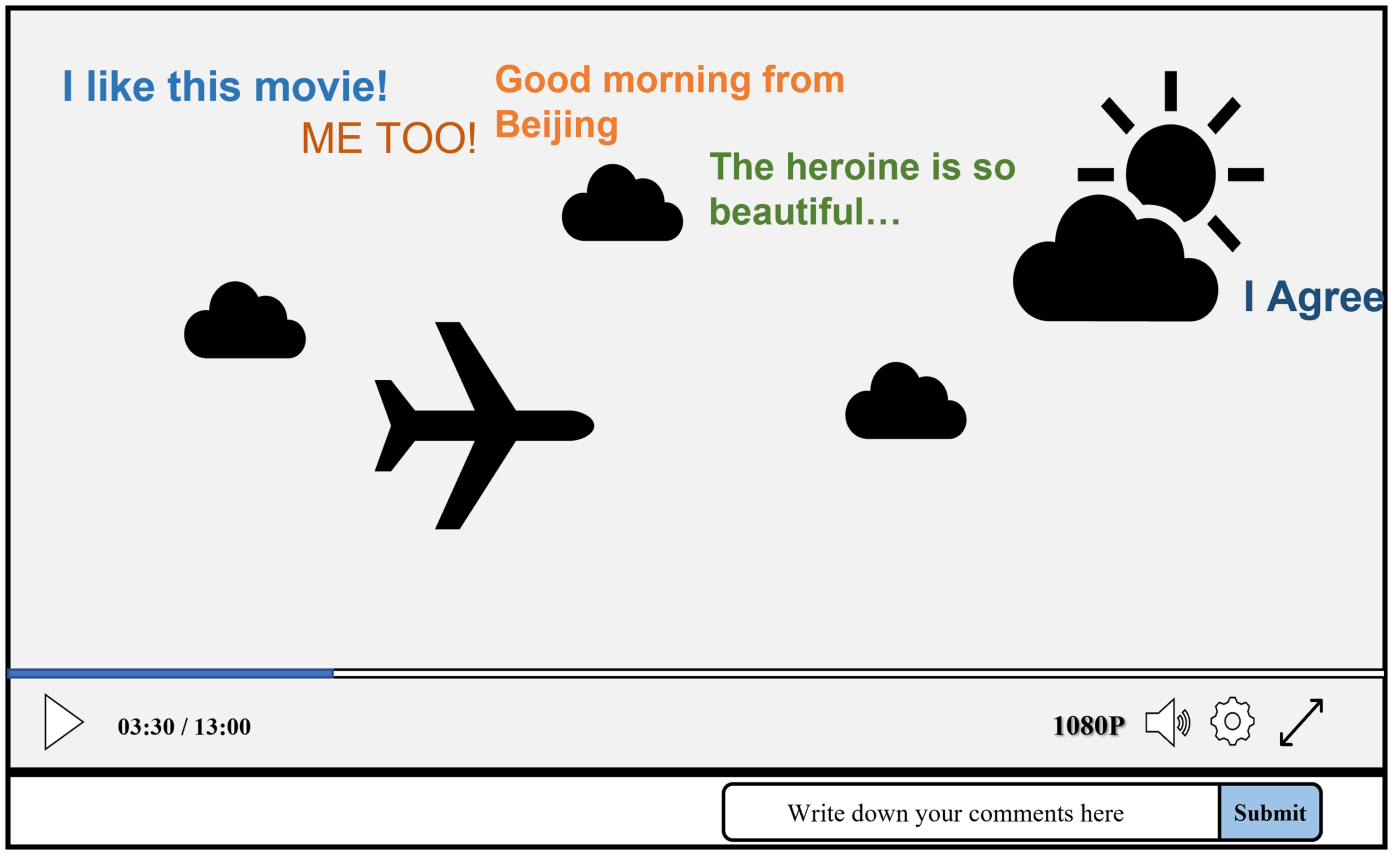
A simplified illustration of Danmu comments – time-synchronic comments laid on top of a video. For a video illustration, see for instance https://www.bilibili.com/video/BV1iK4y177KC/?spm_id_from=333.337.search-card.all.click&vd_source=f9dd652a552053638a9841640a6e3fda (comments on the short documentary film “The Arrival of a Train at La Ciotat Station” directed by Auguste and Louis Lumière).

Recently, a new type of movie comment has emerged in East Asia, notably China. “Danmu” is the Chinese Pinyin for this kind of comment, which is interchangeable with “time-synchronic comment.” Unlike traditional comments arranged under a video, a time-synchronic comment is laid directly on top of it, with synchronization to video playing time (He et al., 2017; see Figure 1). Viewers can thus directly see others’ comments. The advantage of studying time-synchronic comments is the synchronicity, which unravels viewers’ immediate reactions to an on-screen event, and, consequently, the access to how an audience shifts their focus during an unfolding scenario.

In this paper, we focus on feature movies with the following research questions:

1. What is the content of the viewers’ comments, i.e., what are their linguistic characteristics, what are they about and what do they reveal of the viewers’ state of mind?
2. Can viewers’ comments reveal patterns of engagement or immersion *through time*?
3. How can we account for viewers’ patterns of engagement?

After a literature review in Section 2, we introduce the components of our methodology in Section 3, before reporting our results and discussing them in Sections 4 and 5.

## Related work

We focus below on recent studies about live comments and time-synchronic comments. We also introduce the notion of *narrative engagement* at the center of our approach.

Despite the similarity in how video platform users interact with each other while enjoying the same video, time-synchronic comments differ from live streaming ones as they do not require “liveness.” When viewing a video previously recorded, the audience then shares a *pseudo synchronicity* (Zhang and Cassany, 2020) rather than a real one. We review studies on both types of comments to pinpoint the gap in research more comprehensively.

### Live streaming and live streaming comments

Live streaming is a service that can offer real-time human interaction between the streamer and viewers’ while broadcasting an event to an audience online (Hilvert-Bruce et al., 2018). Some live streaming platforms (e.g., Facebook, Twitch, YouTube Live, and TikTok) provide, in particular, an instant messaging interface.

Ample research has been conducted on live streaming platforms. The field of study focuses mainly on a set of particular streaming objects/topics, such as Cricket World Cup competitions (Banerjee, 2018), gaming (Seering et al., 2017; Xu et al., 2021) or news (Luo et al., 2020). Scholars also investigate viewers’ motivations for live streaming engagement (Hilvert-Bruce et al., 2018; Lu et al., 2018), or the impact of live streaming on commerce from both the sellers’ and the buyers’ perspectives (Wongkitrungrueng et al., 2020; Xu et al., 2020; Lin et al., 2021).

Interviews, surveys, and quantitative analyses of aspects of live streaming (e.g., duration or number of viewers) are the main approaches adopted. For instance, Hilvert-Bruce et al. (2018) conducted an online self-report survey of 2,227 Twitch users and utilized linear regression models to identify six motivations that could explain live streaming engagement, which are “social interaction, sense of community, meeting new people, entertainment, information seeking, and a lack of external support in real life” (Hilvert-Bruce et al., 2018). Lu et al. (2018) surveyed 527 live streaming users about their most engaging events in watching and broadcasting experience, and interviewed 14 users to explore their motivations. Wang and Li (2020) crawled information from 1,090 live rooms from the Huajiao platform^1^ and found that commenting was affected mainly by the number of viewers, the gender of streamers, the number of likes and gifts, and the duration of the live stream.

The content of live streaming comments is less investigated. Chen et al. (2021) designed a text-mining-based framework to determine the critical factors in predicting the number of viewers during live streaming events on Twitch. They applied Support Vector Machines, Lasso regression, and K-means clustering to extract the key concepts people talk about during game live streaming. They found that game instruction, game content, appreciation, interaction with the live streamer, game characters, and game time are the six topics game viewers are most interested in.

### Time-synchronic comments

Time-synchronic comments were first used on the Japanese video platform Niconico (Li, 2017) and were then implemented by various Chinese video platforms such as Bilibili, AcFun, Tencent Video, and Iqiyi. Unlike live-streaming events, viewers can stop the video at any specific time point and send a message. In this context, non-live videos such as movies are accessible for time-synchronic commenting after uploading onto the video platform.

Research on time-synchronic comments is done from different perspectives, namely pragmatics (Zhou, 2015; Ruxin, 2018; Wang, 2021; Zhang and Cassany, 2021), communication and media studies (Liu et al., 2016; Zhu, 2017; Zhang and Cassany, 2019; Yang, 2021; Wang, 2022), and computer science (He et al., 2016; Sun et al., 2016; Wang et al., 2020; Zhang et al., 2020).

On the one hand, linguists are curious about the pragmatical functions and cross-cultural distinctions revealed by time-synchronic comments. Wang et al. (2020) explored for example, how and why people are impolite in time-synchronic comments. The author manually collected 26,875 comments on 49 videos that contained impolite metapragmatic comments, and found seven impoliteness strategies. Zhang and Cassany (2019) conducted a corpus study of 1,590 comments on a Spanish TV series, “The Ministry of Time.” They manually labeled viewers’ most discussed topics to study intercultural and sociolinguistic differences between Spain and China. In 2021, they further collected 327 user-selected humorous screenshots of time-synchronic comments to identify the main mechanisms of humor, such as making fun of the plot, of characters, and of each other (Zhang and Cassany, 2021).

On the other hand, scholars from the field of communication and media studies care more about the communicative functions of time-synchronic comments. Liu et al. (2016), adopting the media synchronicity theory, hypothesized that time-synchronic comments as a medium elicit increased interactivity among users. Yang (2021) conducted a social semiotics-oriented multimodal discourse analysis on untranslated English documentaries on Bilibili. He revealed a particular cooperative usage of time-synchronic comments to render Chinese translations for untranslated English videos. Recently, a case study on Russian President Putin-related videos on Bilibili concluded that interaction between time-synchronic commenters promotes community building, and that the collective commenting experience on Bilibili enhances users’ sense of membership (Wang, 2022).

Data-driven approaches are increasingly used to uncover large-scale social/psychological phenomena. He et al. (2017) analyzed 6,506 videos from acfun.tv and found a herding effect and multiple-burst phenomena in time-synchronic comments. To predict the specific comment which contributes most within bursts, they proposed an approach based on comment similarity, comment novelty, comment position, user information, and time information. Furthermore, scholars have discovered the value of time-synchronic comments as a resource for NLP tasks. For instance, Sun et al. (2016) designed *VideoForest*, a system that can convert an input video with time-synchronic comment data into a tree-like visual summary of movie content. As for Wang et al. (2020), they utilized a Bidirectional Long Short-Term Memory (Bi-LSTM) model to classify four emotions – Pleasure, Anger, Sorrow, and Joy – in time-synchronic comments from the NBA (The National Basketball Association) videos available on Tencent Video.

Time-synchronic comments of movies have seldom been investigated, with the notable exception of Zhang et al. (2020). They devised what they called “Moment-to-Moment Synchronicity” (MTMS) as a measure of movie viewers’ engagement during movie consumption (Zhang et al., 2020). MTMS measures the level of synchronicity between a movie’s volume of time-synchronic comments over time and temporal change in its content. Specifically, the authors considered average shot length, number of spoken lines, sound loudness, sound pitch, and motion level as predictors in a regression model to predict the volume of comments, and used the effect size of the prediction as a measure of MTMS. A positive correlation was further found between MTMS and audience ratings of the movies. The study also considered the effect of genre: MTMS is more pronounced in horror and thriller movies, in which suspense is the key element.

All the studies above reveal the potential of time-synchronic comments. However, the rareness of studies on time-synchronic comments left during movie viewing suggests the relevance of a large-scale analysis of comments written during that particular aesthetic process. Our paper focuses on how to fill this gap and uncover movie viewers’ psychological patterns.

### Narrative engagement

When enjoying a movie, the audience may experience disappointment if the main protagonist fails or become angry if s/he is maltreated, as they unconsciously identify with her/him (Trabasso and Chung, 2004). However, to what extent the audience is absorbed, how this immersion evolves through time, and before all, how to measure the immersion in film viewing have long been a question by both moviemakers and psychologists (Visch et al., 2010; Wilken and Kruger, 2016).

To what extent an audience’s attention is grasped by a narrative process is *narrative engagement*, an umbrella word encompassing the feelings of empathy and sympathy, cognitive perspective taking, loss of sense of time, loss of self-awareness, etc. (Busselle and Bilandzic, 2009), when watching movies or reading stories.

Different methodologies and epistemologies can be considered to approach and study narrative engagement. For instance, the method Busselle and Bilandzic (*ibid*) relied upon was to measure the audience’s attentional focus and viewing experience by mean of self-report. Another perspective is the one of neuroscience, where one pays attention to brain processes when an audience perceives narrative messages (Grall et al., 2021). For instance, shared brain responses toward movie narratives can be highlighted by inter-subject correlation (ISC) using functional Magnetic Resonance Imaging (fMRI) across brain regions (Schmälzle and Grall, 2020; Grall et al., 2021). While it might seem easier to collect self-reports than recording brain responses when dealing with many subjects, large-scale approaches are getting more common in neuroimaging, with for instance nearly 350 subjects for the “Narrative” fMRI dataset of brain activity when listening to spoken stories (Nastase et al., 2021).

With the idea that complementary viewpoints can interact fruitfully, we consider a third approach to study a wide audience of viewers: time-synchronic comments, which we argue can reveal instant engagement and reactions much like brain signal and fMRI recordings do, although at a very different level. They appear as promising to not only study an aggregate reaction toward a specific piece of work, but also to follow the evolution of viewer’s engagement through a long period of time, across diverse genres of movies, with a data-driven strategy.

## Methodology

This study has been reviewed and approved by the *Human Research Ethics Committee* (HREC) of *The University of Hong Kong* (Reference number: EA2006009). Data consist of public comments on an online video-streaming platform, without any possible identification of the person – or of their age, gender, location etc. – who published them (beyond a user ID, which we discarded in our analyses).

### Data collection of time-synchronic movie comments

We collected time-synchronic comments in Chinese from a video-streaming platform: Tencent Video.^2^ Most of the movies there are paid content, which means only users who pay for a given movie – or for unlimited access – can watch it and send out comments. We wrote a Python script to crawl 3,831,222 comments from 240 movies. Each time-synchronic comment contains the user ID of the commenter, the calendar time when it was sent, the video time it corresponds to, and its content. We kept only the video time and content of each comment. We deleted non-Chinese characters to minimize noise in the data. Some numbers have a special meaning on Chinese social media, for instance “2333” – when something is amusing – or “6666,” which means “cool” / “amazing.” However, as such significations were not covered by our main analytical tool (the *Simplified Chinese version of the Linguistic Inquiry and Word Count* or SCLIWC, see below), we chose to discard them. After data cleaning, the total number of comments was 3,220,079. The code written to conduct our research and sample time-synchronic comments, as well as a number of outputs, are available in a GitHub repository.^3^

To better understand the effect of time on viewers’ responses, we segmented the comments of each movie into 50 consecutive subgroups, each corresponding to the audience’s comments when viewing 2% of the movie. This segmentation does not accurately divide film narratives according to setup, complications, climax, or epilog (which would have been much more difficult to achieve); however, it allows us to track the chronological changes in viewers’ commenting behavior and underlying evolving psychological activity.

To understand the effect of the movie genre on viewers’ reactions, we chose to study 12 of the genres of the classification provided by the *Internet Movie Database* (IMDB for short).^4^ These 12 genres are **Action**, **Adventure**, **Crime**, **Comedy**, **Drama**, **Fantasy**, **Horror**, **Mystery**, **Romance**, **SciFi**, **Thriller**, and **War**. For each genre, we selected 20 movies from IMDB TOP250 most rated movies within that genre, to ensure that each movie was of significant popularity, for the less popular movies may have had fewer viewers, resulting in a lack of ample comments for analysis. We moved down each list, starting from the most popular movies and skipping those unavailable on Tencent Video. Many movies actually fall into two or three of the genres we chose to study. We thus counted every movie one to three times, which resulted in different numbers of movies eventually falling into each category. We finally collected ratings for each movie in our dataset from both IMDB and Tencent Video (Full list of movies is provided as Supplementary Material S1), and the year in which the movie was produced. Descriptive statistics and examples of movies are listed in Table 1.

**Table 1:** Basic descriptive statistics of collected movies and of their time-synchronic comments.

Movie genre	#Movies	Avg. #comments per movie (rounded)	Avg. #movie rating (IMDB)	Examples of movies
Action	62	22,930	7.37	*Die Hard*, *Captain America*, *The Dark Knight*
Comedy	42	10,357	7.54	*Home Alone*, *Three Idiots*, *Knives Out*
Crime	30	11,978	7.92	*The Godfather I*, *Catch Me if You Can*, *Pulp Fiction*
Drama	137	14,739	7.83	*Forrest Gump*, *The Shawshank Redemption*, *Green Book*
Fantasy	37	17,720	7.40	*Big Fish*, *The Prestige*, *Aquaman*
Horror	27	18,596	7.15	*The Shining*, *Orphan*, *The Conjuring*
Romance	44	13,534	7.63	*Titanic*, *Roman Holiday*, *500 Days of Summer*
Sci-Fi	55	19,403	7.49	*The Matrix*, *BladeRunner*, *Tenet*
Thriller	49	11,596	7.48	*Black Swan*, *The Butterfly Effect*, *Gone Girl*
War	25	16,751	7.87	*Hacksaw Ridge*, *The Flowers of War*, *Saving Private Ryan*
Mystery	50	11,169	7.63	*Vertigo*, *Sherlock Holmes*, *Shutter Island*
Adventure	58	22,922	7.49	*Alice In Wonderland*, *The Chronicles Of Narnia*, *Life of Pi*

### Synchronic analysis of the comments

#### Identifying linguistic and psychological aspects of the comments

To identify the linguistics features viewers’ comments as well as the psychological dimensions they point to, we relied on the *Simplified Chinese version of the Linguistic Inquiry and Word Count* (SCLIWC) software (Gao et al., 2013).

The *Linguistic Inquiry and Word Count* system (LIWC; Pennebaker et al., 2007) is a transparent text analysis program that counts words in linguistically or psychologically meaningful categories (Tausczik and Pennebaker, 2010). The 2007 version of the LIWC system contains a dictionary with words falling into 80 categories (Tausczik and Pennebaker, 2010; Zhao et al., 2016), and the SCLIWC expands it into 102 categories to reflect the special characteristics of Chinese. For example, auxiliary verbs contain words such as “am,” “will,” and “have,” while the category of prepositions gathers words such as “to,” “which,” and “above.” Other categories, such as emotional words, which are more subjective, were built by human judges who decided which words could be suitably included (Tausczik and Pennebaker, 2010). For a given document, the prevalence of each category can be calculated with respect to the total number of words.

Compared to the English LIWC system, the Simplified Chinese dictionary contains a simplified version of the traditional Chinese LIWC, which was translated directly from the English words in the LIWC dictionary, as well as categories and Chinese words frequently used on social media for instance, “哈哈哈 (hahaha, which expresses laughter),” “下载 (download),” “星座 (zodiac signs).” This increases the reliability of SCLIWC when investigating the latter (Gao et al., 2013), and matches our data well.

To deal with the large number of categories of SCLIWC, we conducted a Principal Component Analysis (PCA) to reduce the dimensionality and detect structuring dimensions in our data (Wold et al., 1987) while minimizing information loss (Jolliffe and Cadima, 2016).

#### Topic analysis of the comments

The previous aspects of the comments do not fully reveal their content. We therefore also considered a topic analysis to detect what is commonly discussed by viewers.

A common practice for discovering topics in a set of documents is topic modeling (Wallach, 2006). However, short, little-structured, and colloquial time-synchronic comments do not lend themselves well to conventional approaches relying on “bags of words” and global information, such as LDA (latent Dirichlet allocation). To preserve the local information of individual sentences, we used a sentence transformer, sBERT (Reimers and Gurevych, 2019), with a pre-trained multi-lingual model, in order to build sentence embeddings for our comments. Each comment was thus turned into a vector of 384 numerical values capturing its meaning. We considered only comments with more than 3 Chinese characters to focus on full sentences. As working with the entire set of comments was computationally too demanding, we built a sample of 300,000 comments, with 60,000 comments selected randomly for each of 5 time segments (0–20, 20–40, and 40–60% etc.) to investigate time-based content change.

To build semantically related clusters of comments, we first reduced the number of dimensions of the embeddings with UMAP (Uniform Manifold Approximation and Projection; McInnes et al., 2018), as it has been shown to considerably improve the results (Allaoui et al., 2020). We then applied Hierarchical Density-Based Spatial Clustering of Applications with Noise (HDBSCAN; Campello et al., 2013) with hdbscan Python library (McInnes et al., 2017).

We tuned the hyper-parameters of UMAP – the number of dimensions for dimensionality reduction, the size of local neighborhood, and the minimum distance that the points are allowed to pack together – and of HDBSCAN – the minimum size of a cluster and the minimum number of groups – with a Bayesian search to maximize the number of comments in the largest clusters. The best output corresponded to a minimum cluster size of 283 and 134 clusters totaling together 197,635 comments (the other ones being considered as non-clusterable by HDBSCAN).

To assign meaning to the clusters, we built a document-text matrix from the content of the comments and calculated TF-IDF (term frequency–inverse document frequency) scores for the terms with respect to the clusters. We extracted for each topic the words with the highest scores.

### Diachronic analysis of the comments

While the previous investigations are synchronic, the diachrony of the comments is also relevant and may help to reveal viewers’ engagement through time.

#### Extracting trajectories of viewers’ engagement

The results from the synchronic exploration (see section “Linguistic and psychological dimensions of the comments”) led us to consider two dimensions along which to further study our comments: a cognitive dimension related to analytical/reasoning processes, and another related to emotions. We thus decomposed viewers’ narrative engagement into two parts: one involving an emotional response and the other describing the depth of thinking when the viewer ponders and reflects on details of a movie. We refer to these two parts as emotional engagement and cognitive engagement, respectively. We considered their evolution through movie narrative time and looked for prototypical trajectories.

Tausczik and Pennebaker (2010) described utilizing LIWC linguistic features to measure cognitive complexity, which indicates the richness of reasoning and analysis in thinking. They considered four linguistic features that can be signals of complex thought:

1. Prepositions (introducing concrete information such as location).
2. Words expressing cognitive mechanisms (e.g., words expressing insights and causes such as “cause,” “know,” “ought”).
3. Conjunctions (which join several thoughts together).
4. Exclusive words (e.g., “but” or “without,” used when people operate distinctions between categories).

A positive correlation exists between the usage of words in these four categories and language users’ deep-thinking style (Graesser et al., 2004; Pennebaker et al., 2005).

In terms of emotionality, or the extent to which people express emotions rather than “neutral” analysis, the use of emotional words is a simple but effective measurement, which carries information about the most direct personal response to a positive or negative event.

For every movie, we grouped together the time-synchronic comments of each segment and calculated segment-based cognition-related and emotion-related word rates with SCLIWC – as it performs better with larger amounts of text (Zhao et al., 2016). We thus generated aggregate time series for the different movies, which we then standardized – to focus on their internal shape – and smoothed by applying a moving average with a window size equal to 3 – to prevent abrupt variations from masking meaningful evolutionary patterns. We finally applied soft-DTW (soft-dynamic time warping) k-means clustering (Cuturi and Blondel, 2017) to identify groups of similar trajectories. To decide the optimal k for clustering, we relied on two approaches to optimize reliability: (i) the heuristic “elbow-method” applied to the inertia of the clusters (the sum of the squared distance of the samples to their closest cluster center) for different values of k, and (ii) the locally max silhouette score – a measure of how well each object has been classified (Rousseeuw, 1987) –, once again comparing scores for different numbers of clusters. Monotonic trends of the resulting trajectories were assessed with Mann-Kendall tests (Hamed and Rao, 1998).

#### Distribution of the main topics through time

To get another perspective on how comments evolve as a movie unfolds, we considered the main topics addressed by viewers across five successive time slices, each representing 20% of the total movie time. For each movie, and for each time slice, we computed the prevalence of a topic by dividing the number of related comments by the total number of comments in the time slice. We could then calculate the average prevalence – for each topic and each time slide – for all the movies.

Given that some topics are way more represented in the comments than others, it was not easy to visualize the evolution of the different topics with a heatmap of the previous average values (see Figure in Supplementary Material S8). We therefore further adopted a by-topic approach, where we normalized the values of prevalence of a topic across the 5 time slices.

### Accounting for viewers’ patterns of engagement

#### Influence of movie genres

Movie genres may explain in part the content and features of the time-synchronic comments. To investigate their influence, we focused on the four meta-classes of the SCLIWC, namely *Linguistics Processes* (e.g., function words, verbs, and conjunctions), *Psychological Processes* (e.g., affective words, cognitive mechanisms, biological words), *Personal Concerns* (e.g., work, money, leisure, religion, or yet death), and *Spoken Categories* (words of assent, non-fluencies, and fillers) (Tausczik and Pennebaker, 2010). To assess the impact of our 12 genres on the prevalence of these four classes, we conducted a one-way Multivariate Analysis of Variance (MANOVA), followed by tests of between-subjects effects and post-hoc tests.

We additionally looked at the prevalence of the main topics across genres to detect associations, calculating the proportions of these topics in each movie genre.

Finally, we explored whether movie/narrative genre impacts the trajectories of audience engagement. Given that we found two main trajectories for both emotional and cognitive engagement (see below), we built logistic regression models with the two options forming the predicted variable, and movie genres the predictors. Movie genres were coded as several dummy (0/1) variables, rather than as a single multi-level categorical variable, to account for movies belonging to several genres, similar to the approach taken in Zhang et al. (2020). We then considered the age of a movie (by subtracting the year of production of a movie from 2022) and its rating on Tencent Video Platform as additional predictors.

#### Relationship between topics and engagement trajectories

Besides movie genres, another perspective on viewers’ prototypical trajectories of engagement can be provided by the main topics of the comments.

We first computed, for each main topic, the average degree of cognitive complexity and the degree of emotion of the corresponding comments – specifically, with SCLIWC, emotion-related and cognition-related word rates for the comments of each topic. We then relied on the prevalence of the topics in each movie and each of five successive time slices as defined in section 3.3.2. We averaged these values first for the movies in CogCluster1 and then for the movies in CogCluster2. The difference between these two clusters, CogCluster1 and CogCluster2, lies in their ability to provoke the audience’s cognitive engagement through movie time. To be specific, when people are watching movies that are classified into CogCluster1, their comments tend to have an increasing rate of cognition-related words, while movies in CogCluster2 tend to have a decreasing rate. Given that both heatmaps of prevalence of topics looked very similar for the two clusters, we then subtracted the values for CogCluster2 from the values of CogCluster1 to create a contrastive heatmap and observe relative differences between the two clusters. We followed the exact same approach for EmoCluster1 and EmoCluster2, which are similar to the cognitive clusters, with movies in EmoCluster1 stimulating the audience to comment with increasing emotionality, while movies in EmoCluster2 favor decreasing emotionality.

#### Explaining the engagement trajectories by movie narrative

To possibly explain viewers’ engagement with movie content, we collected screenplays for 179 of our 240 movies – not all the screenplays of our movies were accessible. These screenplays were all written in English. Character names and settings of scenes (in the format of “INT.” or “EXT.,” which represent the entering and exiting of a scene) can repeat hundreds of times in a screenplay; they are, however, unrelated to cognitive and emotional aspects, and were discarded. Similar to segmenting comments, the text of each screenplay was chunked into 50 equal segments based on word count – for instance, a 30,000-word screenplay led to 600-word segments. This approach roughly approximated segmentation by movie time, as screenplays do not only include dialogues but also descriptions of characters and of their environment. We then calculated cognition-related and emotion-related word rates for each screenplay segment with the 2007 version of LIWC. The clustering of the resulting time series was the same as for comments.

To assess the relationship between narrative content and narrative engagement, we applied Granger-causality tests (Granger, 1969) to word rates of time-synchronic comments and screenplays. In a nutshell, if the emotional/cognitive trajectory from a movie screenplay (X1) “Granger-causes” the emotional/cognitive trajectory of the audience who watches this movie (X2), then the past information of the movie screenplay (X1) should help predict audience response (X2), and this prediction should be better than the one made with only past values of X2. The time shift between the past and the current time is counted in units of passing time, or lags. For example, if X1 Granger-causes X2 with a lag of 2, it means that the information at the past two time units (t^−2^) of X1 Granger-causes X2 (at t). As a unit of time in our study equals 2% of movie time, we only considered the influence of movie content on viewers within 5 lags or 10% of movie time, to account for viewers’ limited and shifting attention.

To meet the assumptions of Granger-causality tests, we checked time-series stationarity with the Augmented Dickey–Fuller test (Dickey and Fuller, 1979). Non-stationary time series were stationarized by taking their first-order differences ***Δ**(**t**) **= x**(**t**)**-x**(**t-1**)*, which were then also tested for stationarity.

## Results

In the next subsections, we report our results and provide answers to our initial questions: what is the content of the viewers’ comments (in synchrony), what do they reveal in terms of diachronic patterns of engagement, and how can we account for such patterns?

### Synchronic analysis of the comments

#### Linguistic and psychological dimensions of the comments

Upon applying the PCA described in Section 3.1 to our 240 movies, we observed that the first two components accounted for 25.01% of the total variance, with 15.12% of the inertia for the first component and 9.89% for the second. Such a total amount of variability explained is statistically significant (the 0.95-quantile of the inertia percentages distribution generated by simulation of 1,668 data tables of equivalent size with normal distribution is equal to 5.2%).

**Figure 2:**
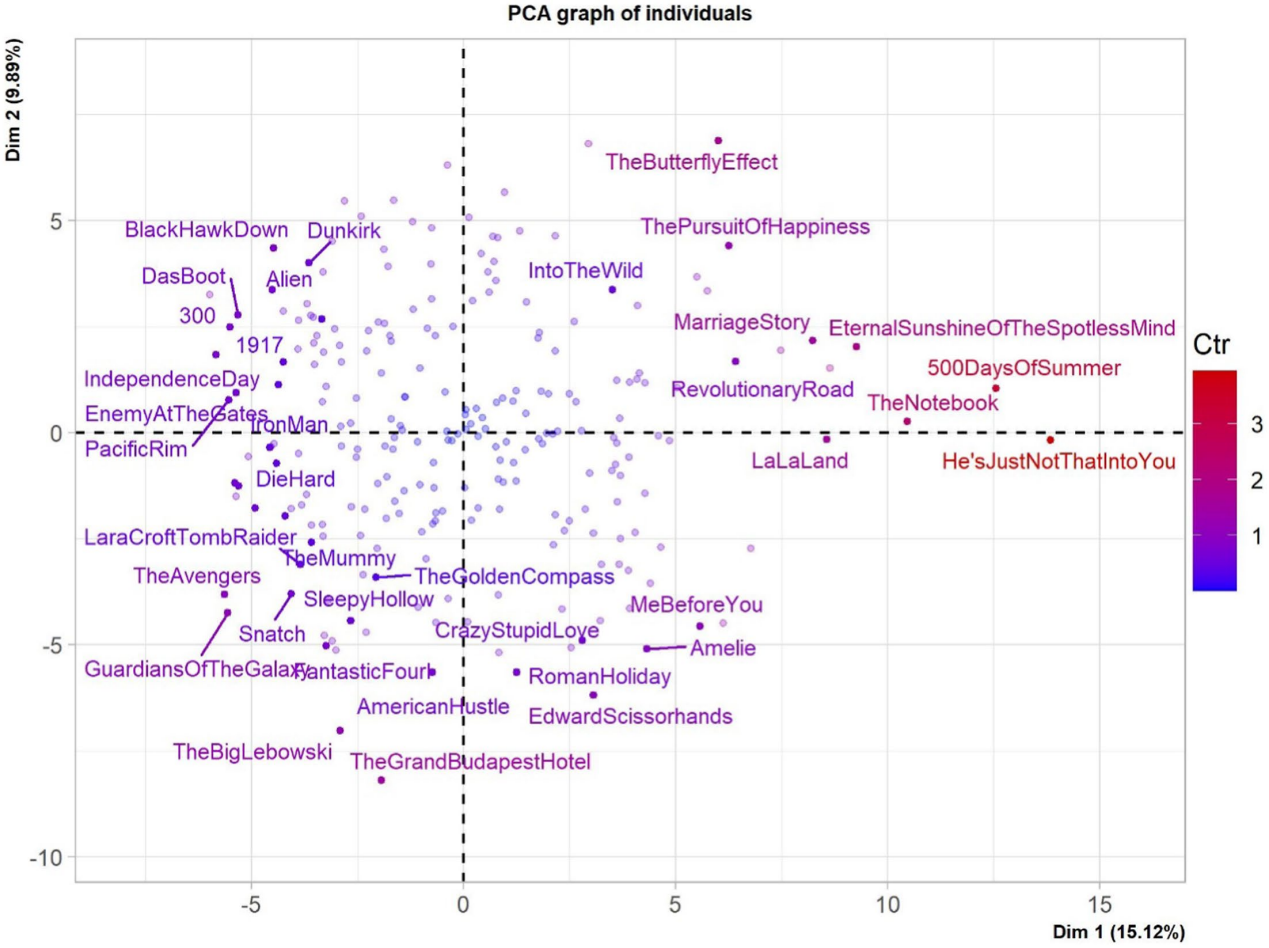
The individual factor map of Components 1 and 2 of the PCA of SCLIWC features for 240 movies. The labeled individuals are those with higher contributions to the plane construction. The *X*-axis is the first principal component (Dimension 1), the *Y*-axis the second principal component (Dimension 2).

**Figure 3:**
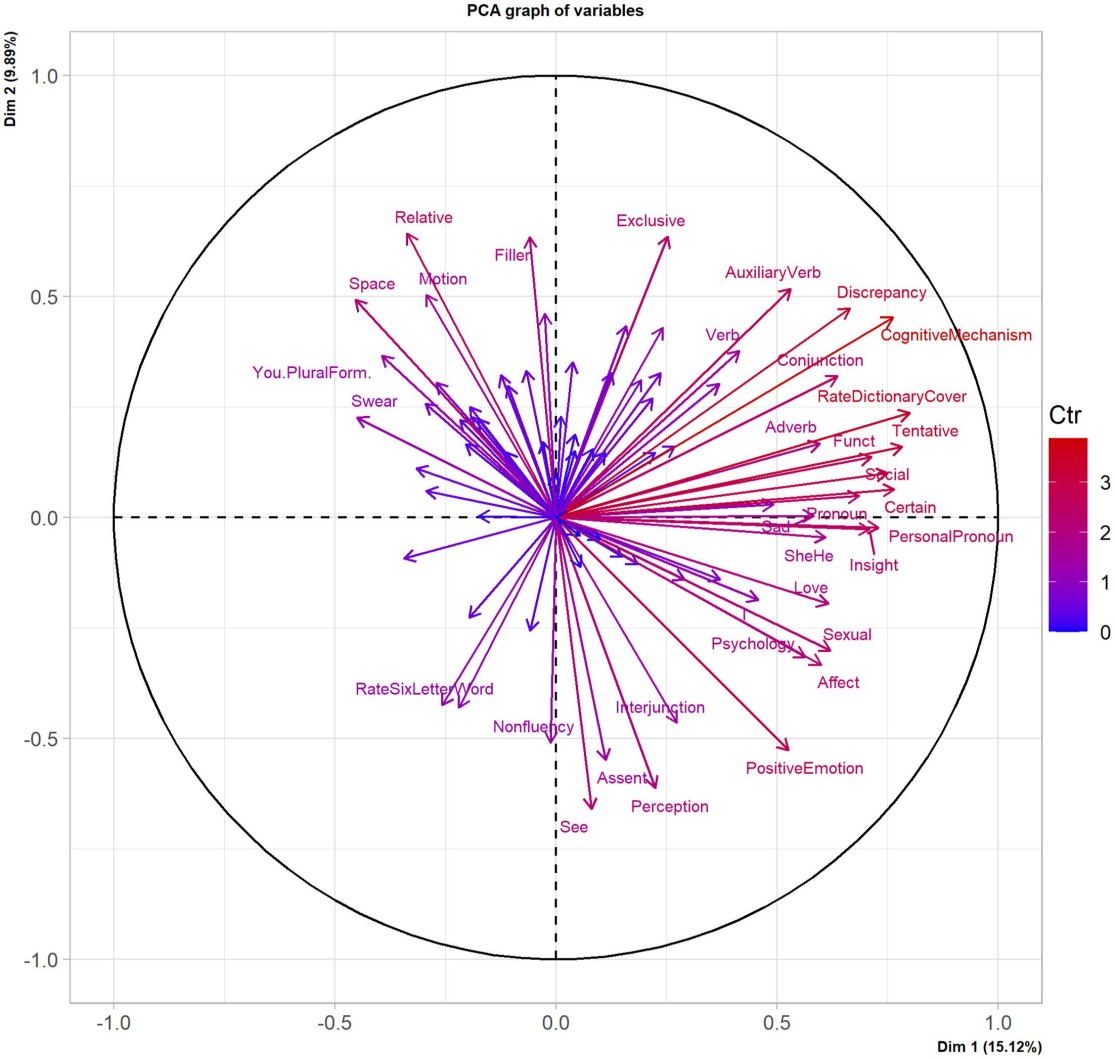
Variables factor map of Components 1 and 2. The labeled variables are those with higher contributions to the plane construction. The *X*-axis is the first principal component (Dimension 1), and the *Y*-axis is the second principal component (Dimension 2). The variables help to interpret the distribution of individuals (i.e., movies) in Figure 2, for instance, individuals in the upper-right corner in Figure 2 are those with high values for the variables positively correlated with the first and second components.

The individual factor map and the variable factor map for the first two components are reported in Figures 2, 3, respectively. Taken together, they allow identifying different quadrants with their movies, and the linguistic and psychological features of their comments. If one pays attention to the four quadrants, one can observe that:

- In the upper-right quadrant, one finds comments of movies which seem mostly characterized by deep meaning and deep interactions among characters – **drama** and **romance** like *The Pursuit of Happiness*, *Into the Wild*, *Eternal Sunshine of the Spotless Mind* etc. Associated SCLIWC variables are, for instance, **Cognitive Mechanisms**, **Social** or **Inhibition** on the psychological side, and **Exclusive**, **Adverb**, **Pronoun**, and **Discrepancy** on the more linguistic side;
- The lower-right quadrant also seems to contain **drama** and **romance** movies, but corresponding SCLIWC variables are this time **Perceptual**, **Positive Emotion**, **Sexual**, **Affect**, **Psychology**, etc. on the psychological side, and **Interjunction** or **I** on the linguistic side;
- In the upper left quadrant, one finds a number of war and SciFi movies such as *300*, *Black Hawk Dawn* or *Independence Day*. Associated SCLIWC variables are **Swear**, **Space**, **Motion**, **Relative**, **Death**, **Anger,** etc.
- In the lower-left quadrant, the comments are mostly those of **Action**, **Comedy**, and **Crime** movies, which contain frequent action sequences, like *The Avengers*, *Guardians of the Galaxy*, *The Big Lebowski* or *The Grand Budapest Hotel*. Associated SCLIWC variables are **Rate of 4-character words** (for instance, “Hahahaha”), **Body**, or yet **Leisure**.

The first dimension appears to oppose features of casual language – fillers, non-fluencies, swearing, “hahahaha” – to those of more formal and elaborated language – adverbs, markers of certainty or discrepancy, etc. When movies are less demanding in comprehension (especially action movies), their comments include more informal words and casual expressions, and *vice-versa*. It makes sense that watching action-filled movies lead to comments reflecting viewers’ “shallower” entertainment both in terms of content and linguistic surface.

The second dimension involves variables that relate more to the mental states of the audience. While negative values seem to relate more to emotions, positive values imply reasoning and reflection. Watching movies that depict quests for meaning, social relationships and emotions leads to comments loaded with affects and psychological processes. Suspense and mystery similarly involve reasoning, and the language of the comments shows viewers’ attempts to find out the truth buried in chains of events.

A take-away from this first analysis is that the influence of movies on viewers’ mental states is at least two-pronged, with emotions on the one hand and reasoning on the other. A suggestion is that the second dimension opposes variables which show how the audience is dragged into the fictional world (experiencing emotionally), to how the audience remains out of this world, in order to analyze it and come up with explanations. Such a distinction can be further exploited diachronically – i.e., as movies unfold –, as we will see in later sections.

#### Main topics addressed in the comments

The method described in 3.2.2 led us to identify 15 main topics (those with the most comments) – out of a total of 134 topics – for the 300,000 randomly selected comments. For each of them, the words with the highest TF-IDF scores help to provide a general description of the content. Cluster names/identifiers, cluster overall content, corresponding words with the highest TF-IDF scores in each cluster and counts of related comments are provided in Table 2. In total, 104,546 comments appear in these 15 clusters. Examples of comments in each cluster are provided in Supplementary Material S2.

**Table 2:** Top-15 clusters and words with the highest TF-IDF scores in each cluster.

Cluster number	Topic	Words with highest TF-IDF (English translation)	Count
128	Audience interaction	*Could not bear*, *heart*, *meaning*, *foolish*	27,845
112	Female characters	*Princess*, *queen*, *girl*, *sister*	19,028
37	Movie genre-movie production	*Cinema*, *sci-fi*, *old movie*, *horror movie*, *classic movie*	7,745
124	Death	*Murderer*, *suicide*, *kill*, *died*, *killer*	7,267
26	China-Country	*China*, *Chinese*, *Shanghai*, *Beijing*, *Japan*	5,834
123	Male characters	*Gentleman*, *lie*, *male character*, *self-abased*	5,596
132	Audience check-in	*Chongqing*, *Guangdong*, *Hunan*, *Guangxi*	5,576
118	Parent–child Relationship	*Boy*, *father*, *childhood*, *parent’s love*	5,344
106	Money	*Rich people*, *Renminbi*, *pay money*, *loterry*, *cost*	3,715
77	War-military	*War*, *soldier*, *war movie*, *battlefield*, *World Wars*	3,689
99	Food	*Delicious*, *spicy strips*, *steak*, *pepper*, *beef*, *bread*	2,844
10	Amusement	*Laughter*, *comedy*, *haha*, *humorous*, *amusing*	2,680
109	Marriage-family	*Wife*, *marry*, *divorce*, *marriage*, *husband*	2,572
127	Favorable comments on movies	*Fabulous*, *good*, *brilliant*, *inspiring*, *worth watching*	2,522
86	Fear	*Fear*, *afraid of*, *phobia*, *scary*	2,288
		**Total**	**104,545**

**Figure 4:**
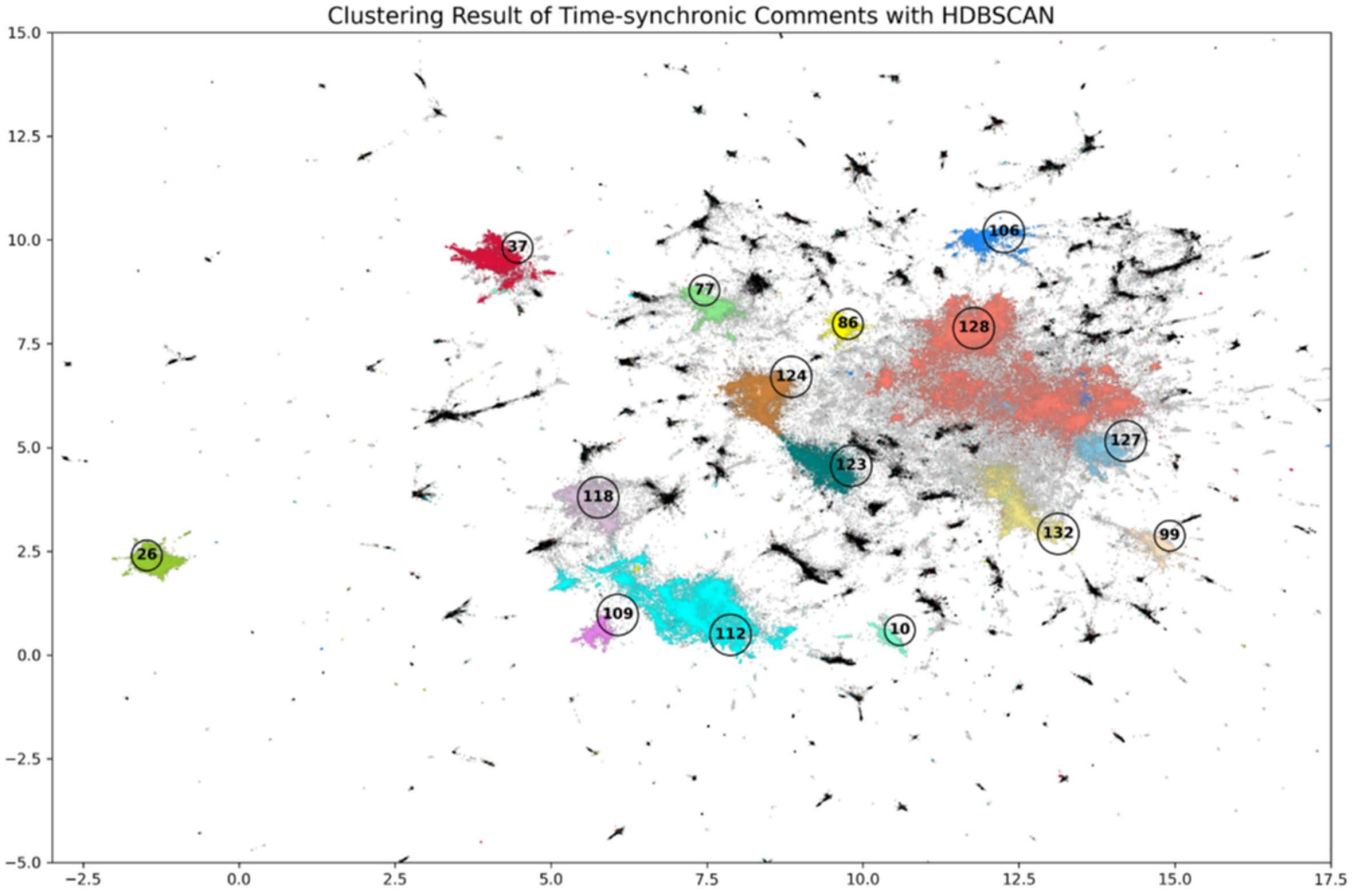
HDBSCAN outputs for the time-synchronic comments projected in a 2-D space with UMAP. The top 15 clusters are colored and labeled. The x-axis and y-axis are the two dimensions on which the sentence embeddings are projected. Cluster numbers can be found in Table 2.

For the sake of illustration, we projected the result of the clustering into a 2-D space, once again using UMAP to go from 384 to 2 dimensions. The resulting structures can be seen in Figure 4 with labels and colors.

It appears first of all that the viewers interact a lot with each other (***Cluster 128***), with 26% of the total amount of comments being related to these interactions. Through **interactions**, they argue about the movie content, express their confusion when watching, etc., and wait for other viewers to answer their questions (e.g., “*Can anyone tell me why Captain American still does not appear till now?*”). The second topic which is discussed most is **female characters** (***Cluster 112***). It seems that the audience cares much about female characters’ external appearance (e.g., “*The heroine is so beautiful*.”), their behaviors (e.g., “*Her action is really hypocritical*.”), and the actresses who star as these characters (e.g., “*She is Angelina Jolie’s daughter*.”). The audience also comments quite frequently on **the production and genre of movies** (e.g., “*This is the first horror movie I watched when I was 6 years old*.”), **death** (e.g., “*They will all die in the end*.”), **China or China’s relationship with other countries** (e.g., “*China and Japan had a war at that time*.”), and **male characters** (e.g., “*The male character cares about nothing but benefits*.”). Furthermore, the online platform provides viewers with a place to meet and share their viewing experience, leading to a large number of **audience check-ins**, which usually include the viewer’s location (Liu et al., 2014). For instance, a viewer may write something like “*I*, *who is from Chongqing*, *finished the movie today*.” to wrap up the movie, or leave a sentence such as “*Hello everyone! I’m from Guangzhou*.” as a greeting at the beginning. Finally, **parent–child relationships**, **money**, **the military**, **food**, **amusement**, **family or marriage**, **supportive comments for a specific movie** or character, and **fear** also make up for many comments and therefore reveal where viewers’ attention goes.

Overall, even without going further in the analysis of smaller clusters, one cannot but observe the diversity of the audience’s interests.

### Diachronic analysis of the comments

#### Prototypical trajectories of viewers’ emotional and cognitive engagement

How does viewers’ emotional and cognitive engagement evolve through time in film viewing? Building and comparing the trajectories of emotional and cognitive engagement for our different movies, we found that they can be best described as falling into two clusters/categories – put differently, the optimal k for k-mean clustering appears to be 2 for both emotional trajectories and cognitive trajectories. We call them ***EmoCluster1***, ***EmoCluster2***, ***CogCluster1*** and ***CogCluster2*, *respectively*.** The results of the clustering of movie comments are provided in Supplementary Materials S3, S4.

**Figure 5:**
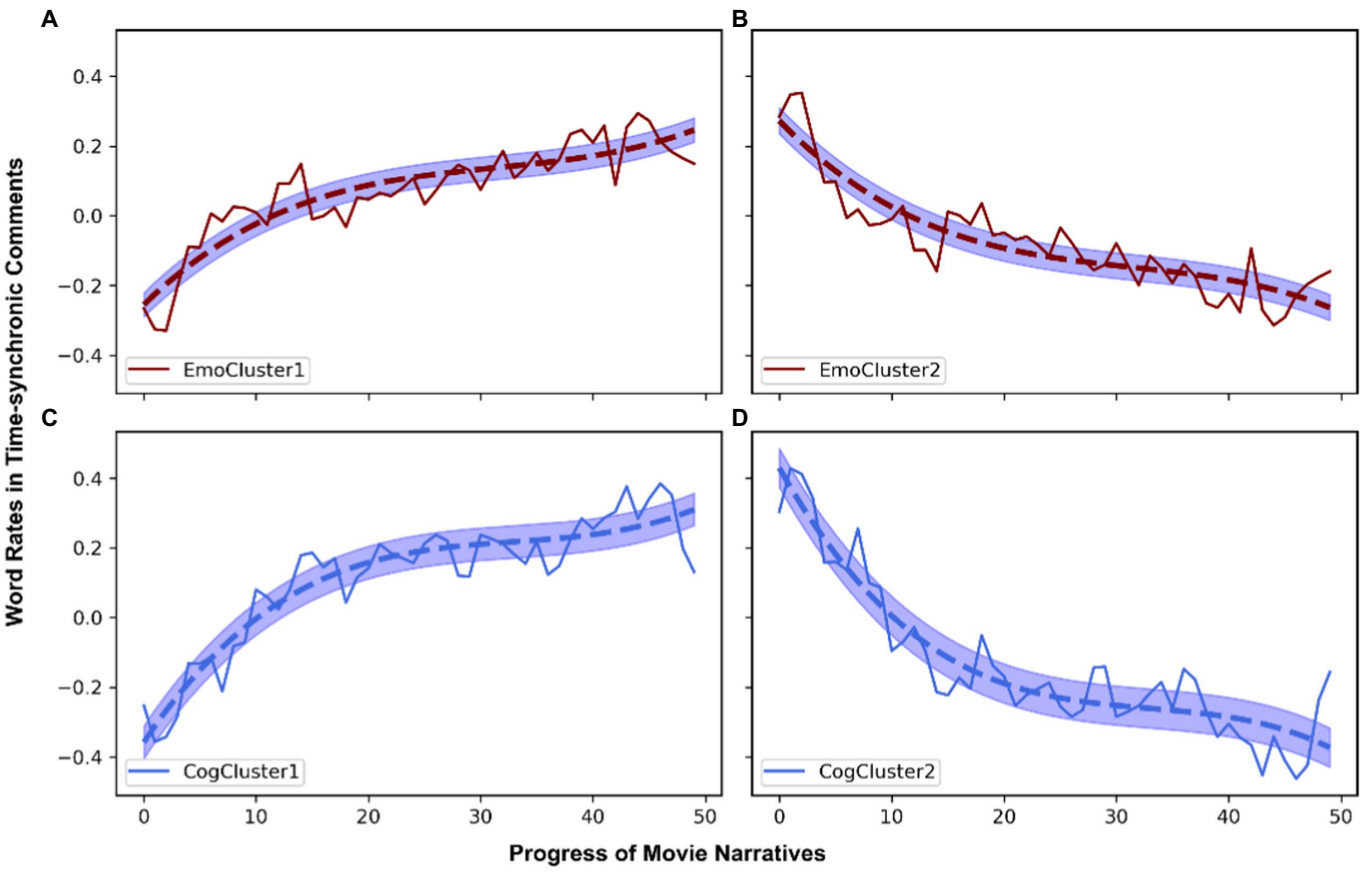
Aggregate time series, i.e., prototypical trajectories, of evolving emotional **(A,B)** and cognitive engagement **(C,D)** in comments, as generated with soft-DTW k-means clustering. The *X*-axis represents 50 time-slices of the movie narrative (each corresponding to 2% of movie time). The *Y*-axis shows the standardized proportion of emotion-related (upper panels) or cognition-related (lower panels) words used in the comments.

Figure 5 shows the four prototypical trajectories. All are monotonic (*p* < 0.05).

The first trajectory of cognitive engagement, which represents the averaged cognition-related word rates in the time-synchronic comments of 131 movies across narrative time (***CogCluster1***), shows an obvious upward trend, though with fluctuations. When watching these movies, the audience gets more cognitively engaged as they unfold. On the other hand, the second “cognitive trajectory,” which is derived by averaging the trajectories of 109 movies (***CogCluster2***), displays a downward trend: as they watch these movies, viewers’ comments suggest that they devote less and less cognitive resources.

As for emotional engagement through time, the 124 movies classified into ***EmoCluster1*** tend to see viewers undergo an increasingly intense emotional experience, as the increasing trend implies. On the contrary, the 116 movies in ***EmoCluster2*** see decreasing use of emotional resources for comprehension.

In a nutshell, both cognitive and emotional engagements through time appear to be quite binary at a macro-level: either increasing or decreasing. These two modes of engagement are also independent from each other. What underlies this organization requires further analysis, and a plausible explanation lies of course in the content of the movies, as analyzed further in 4.3 and 4.4.

#### Distribution of the main topics through time

**Figure 6:**
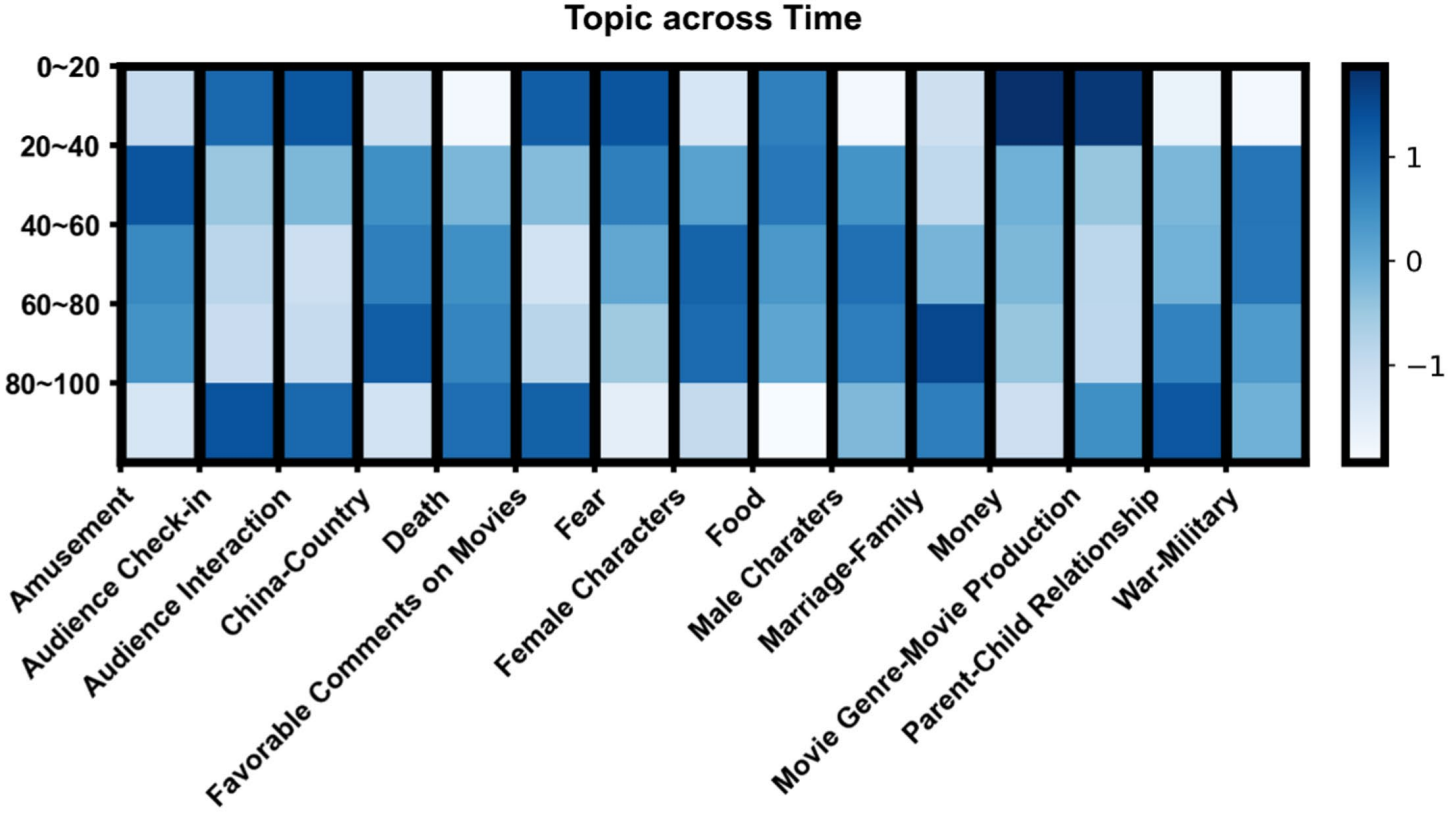
Normalized distribution of the 15 main topics across time, with narrative time divided into five successive equal-length segments. The vertical bars indicate that color variations should be considered column-wise only, and not row-wise, because of the normalization process to better visualize the diachronic profile of each topic.

Figure 6 displays the normalized prevalence of the main topics of the comments across 5 successive time slices for the movies: 0–20, 20–40, 40–60, 60–80, and 80–100% of the total movie time.

Some interesting patterns can be, noticed. At the beginning of the movies (0–20% of the movie narrative time), viewers tend to **check in**, talk about how they like this movie, and tell others how much money they paid to the video platform for movie watching, their **fear** in connection the movie setting and atmosphere, and whether the movie is of a specific **genre**. As the movie unfolds (20–80%), the distribution appears to be more balanced – the medium band of the heatmap appears to be less contrasted –, with possibly people’s attention attracted to genre-specific content (to be assessed in 4.3.2). Toward the end (80–100%), some topics appear to be discussed more intensively, with once again **checks-in**, comments about **death** (of the characters) or **movie genre/production**, and **favorable comments** toward the movie and the producer. **Parent–child relationships** also remain of interest, with both positive comments like “*The father really respected his son*,” or negative ones, e.g., “*I cannot understand why the parents adopted the girl when they already had their own children*.”

While **fear** was an important topic at the beginning, this is no longer the case at the end. This topic is actually discussed less and less as a movie unfolds, suggesting that the audience gets over this emotion. A different pattern appears for **amusement**, which is expressed more in the middle of the movies. The opposite distribution is observed for comments on **movie genre/production** and **checks-in**.

All in all, while some patterns can be expected, such as when it comes to **checks-in** or **fear**, the distribution of topics through time does not present a very high degree of structure, suggesting the need for additional explanatory variables.

### Explaining viewers’ engagement

#### Distribution of linguistic and psychological dimensions across movie genres

While we partially interpreted the previous PCA with respect to movie genres, we did not investigate the latter with tools from inferential statistics. As establishing relationships between the latter and the many variables of the SCLIWC was rather daunting, we focused on four meta-classes of features: *Linguistics Processes*, *Psychological Processes*, *Personal Concerns*, and *Spoken Categories*.

The MANOVA with the prevalence of the four previous classes and the various genres as predictors reveals a statistically significant overall effect [*F*(44, 2,301) = 7.178, *p* < 0.0001]. Movie genres thus bear a statistically significant impact on the nature of viewers’ time-synchronic comments. In terms of effect size, the partial η^2^ is equal to 0.116, which means that 11.6% of the variance of audiences’ commenting behavior is due to the exposure to different movie genres.

Tests of between-subjects effects help to further determine the impact of the different genres on the four dependent variables considered separately. It appears that movie genre has a statistically significant effect on all *Linguistic Processes* [*F*(11, 604) = 9.067; *p* < 0.0001; part. η^2^ = 0.142], *Psychological Processes* [F(11, 604) = 14.485, *p* < 0.0001; part. η^2^ = 0.209], *Personal Concerns* [F(11, 604) = 4.621; *p* < 0.0001; partial η^2^ = 0.071], and *Spoken Categories* [F(11, 604) = 8.992; *p* < 0.0001; partial η^2^ = 0.141; all *p*-values adjusted with Bonferroni Correction]. The effect size of genre is thus strongest for *Psychological Processes*, and weakest – 3 times less – for *Personal Concerns*.

Tuckey’s post-hoc tests informed us of differences among various pairs of genres in terms of our four classes (see Supplementary Material S5). Many pair comparisons were significant, for instance, while comments of **Action** and **Adventure** movies did not appear to significantly differ in any of the variables, comments for **Romance** movies differed from those of **Crime** movies for *Linguistic Processes*, *Psychological Processes*, and *Personal Concerns*.

There thus appears to be an overall significant effect of movie genre on viewers’ commenting behavior: they experience different psychological movements when exposed to varying types of fictional worlds and events. Effect sizes, however, remain small.

#### Distribution of topics across movie genres

**Figure 7:**
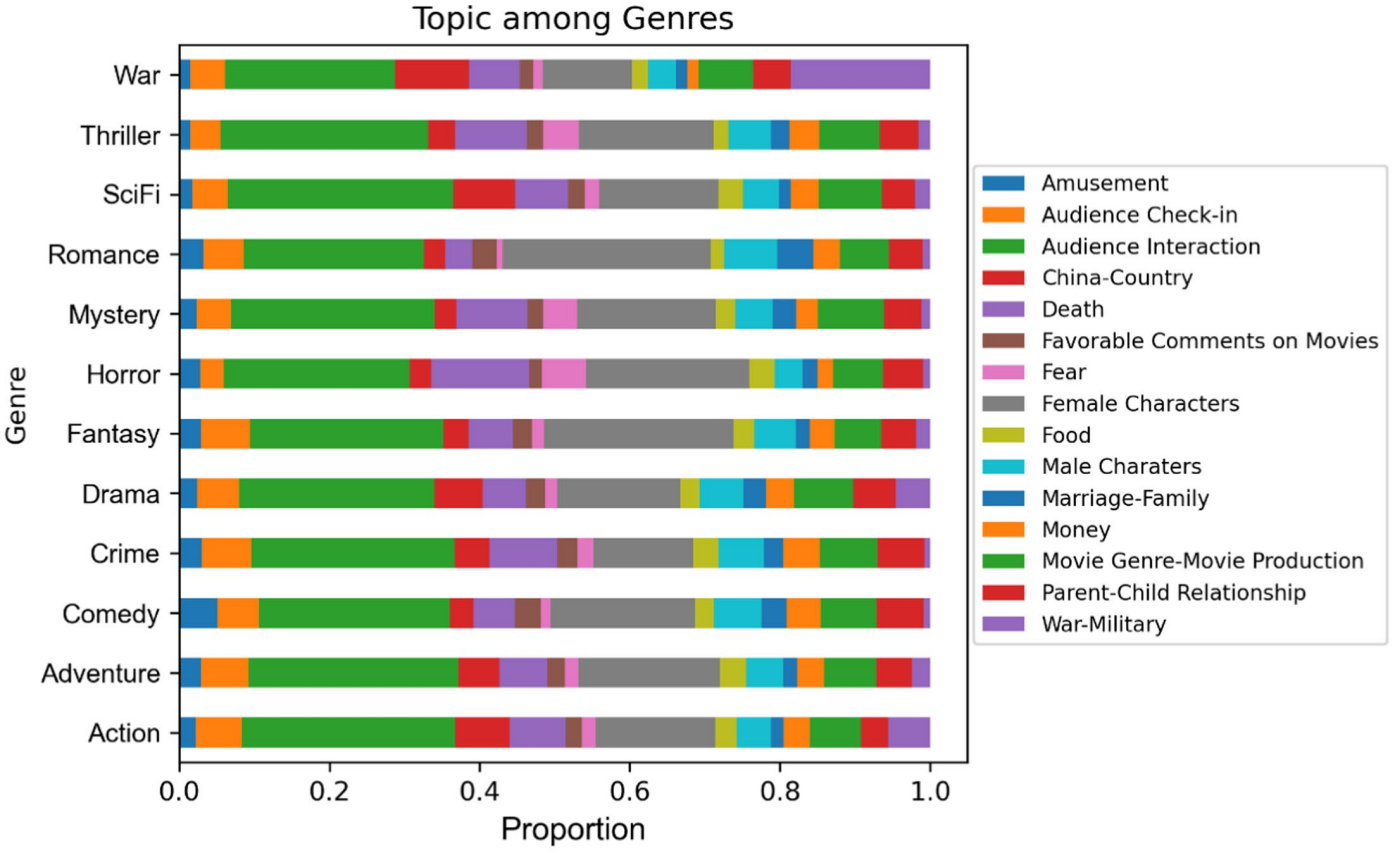
Topic distribution among genres. The topic “China and China’s relationship with other countries” was written as “China-Country” for short. The full graph which contains the exact proportions of each topic among genres can be found in Supplementary Material S9.

Figure 7 depicts the distribution of the main topics among genres.

For all genres, **audience interactions** amount to the largest ratio of comments. The audience also consistently likes to talk about **female characters**, whatever the **genre**.

While, perhaps surprisingly, all the topics appear in all the genres, the distinctions between genres can be captured by some differences in prevalence, most of which are quite expected. One can note in particular that:

- Though **female characters** are always of interest to viewers, this is especially true in **Romance**, **Horror** and **Fantasy** movies, and less so in **Crime** and **War** movies; On the other hand, **male characters** are of special interest to the audience when it is watching **Romance** and **Comedy** but are less discussed in **Horror** and **War** movies.
- When people watch **Crime**, **Horror**, and **Mystery** movies, their attention to **death** is expectedly greater than when watching **Romance**.
- **War and military aspects** are most prevalent in **War** and **Action** movies, and nearly absent in other genres such as **Comedy** or **Romance**.
- Interestingly, **War** and **SciFi** movies attract the most comments on **China and its relationship with other countries.**

On the contrary, some topics are distributed quite equally across all genres, such as **movie genre/production**, **parent–child relationship** or **food**.

Overall, once again, movie genres appear to influence the content of the comments. One could have expected more drastic differences between genres, but effects are nevertheless quite visible.

#### Impact of movie genre, age, and rating on audience engagement

Movie genres may also be considered to account for an audience’s cognitive and emotional engagement trajectory during movie viewing (the number of movies that fall into each cluster can be found for the different genres in Supplementary Material S6). Can they predict which of the two trajectories previously described a movie is following?

We tried to predict the two broad patterns of emotional engagement, and the similar patterns of cognitive engagement, with movie genre, age, and rating. The coefficient estimates and pseudo-R2 of the corresponding regression models appear in Table 3.

**Table 3:** Coefficient estimates and pseudo- *R*^2^ (in parentheses) of the logistic regression models predicting the cognitive or emotional pattern of engagement (i.e., predicting either CogCluster 1 or CogCluster2, and either EmoCluster1 or EmoCluster2).

	Dependent variable			
	CogCluster (a)	EmoCluster(b)	CogCluster(c)	EmoCluster(d)
MovieAge			−0.025^**^ (**0.024**)	0.017 (0.013)
TencentScores			−0.443 (0.006)	−0.478 (0.009)
Crime	−0.662 (0.008)	−0.024 (0.000)	−0.733 (0.009)	−0.011 (0.000)
Drama	−0.708^*^ (**0.014**)	1.075^**^ (**0.034**)	−0.866^*^ (**0.019**)	1.272^***^ (**0.044**)
Action	0.740^*^ (**0.017**)	0.268 (0.003)	0.488 (0.006)	0.332 (0.003)
War	−0.414 (0.003)	0.474 (0.004)	−0.229 (0.001)	0.317 (0.001)
Thriller	−0.406 (0.003)	0.342 (0.003)	−0.582 (0.006)	0.370 (0.003)
Mystery	−0.663 (0.013)	0.230 (0.002)	−0.648 (0.011)	0.152 (0.001)
SciFi	−1.047^**^ (**0.030**)	0.167 (0.001)	−1.242^***^ (**0.038**)	0.194 (0.001)
Adventure	0.572 (0.009)	0.332 (0.003)	0.560 (0.007)	0.455 (0.005)
Comedy	0.093 (0.000)	1.214^**^ (**0.037**)	−0.116 (0.000)	1.313^***^ (**0.040**)
Romance	−0.257 (0.001)	0.083 (0.000)	−0.217 (0.001)	0.050 (0.000)
Fantasy	−1.211^**^ (**0.032**)	0.338 (0.002)	−1.472^***^ (**0.041**)	0.331 (0.002)
Horror	−0.144 (0.000)	−0.630 (0.008)	−0.371 (0.002)	−0.720 (0.010)
Observations	240	240	240	240
Log Likelihood	−149.994	−155.285	−145.912	−153.581
Akaike Inf. Crit.	325.989	336.570	321.824	337.162

The first two columns are results yielded by models with only movie genres as predictors, and the last two consider Movie Age and Tencent Scores as additional predictors. Significant (*p* < 0.05) predictors are made bold.*p < 0.01; **p < 0.01; ***p < 0.01.

Leaving first movie age and rating aside, we found **Science Fiction** and **Fantasy** to be significant predictors (*p* < 0.05) for the pattern of cognitive engagement, while **Drama** and **Action** show a tendency (*p* < 0.1) (column a in Table 3). Positive estimates weigh in favor of ***CogCluster2*** (decreasing engagement), and negative estimates in favor of ***CogCluster1*** (increasing engagement). For instance, a movie being a SciFi movie decreases the likelihood of this movie to belong to ***CogCluster2***/increases the likelihood to belong to ***CogCluster1***. Looking only at significant effects, **Science Fiction** and **Fantasy** therefore both promote increasing cognitive resources for comprehension as the movie unfolds. The effect size remains, however, small, as revealed by the values of the McKelvey-Zavoina Pseudo- *R^2^* (McKelvey and Zavoina, 1975): 0.030 and 0.032, respectively.

As for emotional engagement (column b), only **Drama** and **Comedy** are statistically significant (*p* < 0.05). Positive estimates mean that these two genres both increase the likelihood of experiencing increasingly intense emotions. Effect sizes are very comparable to the previous ones: 0.034 and 0.037, respectively.

When movie rating and age are included as predictors (columns c and d), the former significant effects remain, while the tendency for **Action** disappears. **Age** is significant (*p* < 0.05) and increases the likelihood of an upward trend for cognitive engagement – the older a movie, the more likely the audience will increasingly get engaged cognitively, though the effect size is once again small. No effect is observed for emotional engagement.

In short, our statistical models indicate that only a few movie genres significantly predict engagement trajectories, additionally without much strength. Broad diachronic patterns of engagement might therefore be quite independent from movie content.

#### Connecting topics and engagement trajectories

Movie genres do not explain much of the trajectories of audience engagement. Can topics shed a better light?

**Figure 8:**
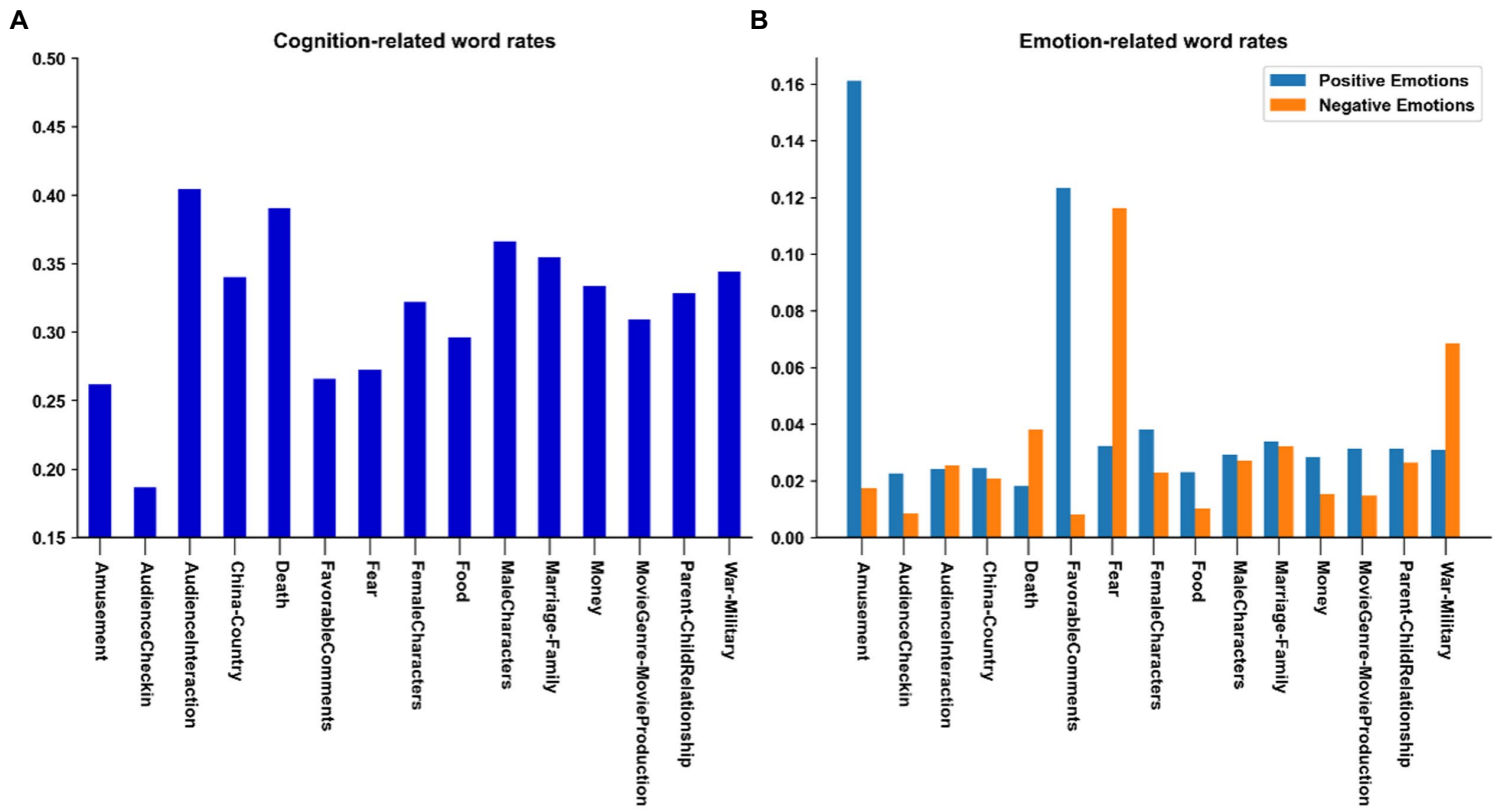
Cognitive complexity **(A)** and emotionality **(B)** of each topic. The *Y*-axes indicate the proportions of cognition-related **(A)** and emotion-related **(B)** words used in the comments.

Figure 8 provides the average cognitive complexity and degree of emotionality of each of the main topics discussed in the comments – as assessed with cognition and emotion-related word rates. With respect to cognition first, we can see that **checking-in** involves the least engagement. On the contrary, audience members devote the most cognitive resources to **interacting** with others – offering them their thoughts on the movie or trying to persuade them (e.g., “*You cannot understand how important the hope is for a man in a dilemma*.,” “*We should not laugh at the character’s action because he really has no choice but to do that*.”). **Death** also promotes reflection while **fear** tends to inhibit it, which indicates that **death** and **fear** do not necessarily go together. For instance, when watching a murder mystery, viewers will talk about the death of a character without being afraid of it. As for emotional engagement, we considered polarity for further insight, and observed expectable results: the proportion of positive emotions is high in **amusement** (***Cluster 10***) and **favorable comments on movies** (***Cluster 127***), while **fear** (***Cluster 86***), **war-military** (***Cluster 77***), and **death** (***Cluster 124***) evoke way more negative emotions than positive ones.

One might now formulate the following hypothesis: the prototypical engagement trajectories may be explained by the contrasted prevalence through time of some key topics in terms of cognitive and emotional complexity – something not clearly enough revealed by movie genres given the complex relationship between genres and topics.

**Figure 9:**
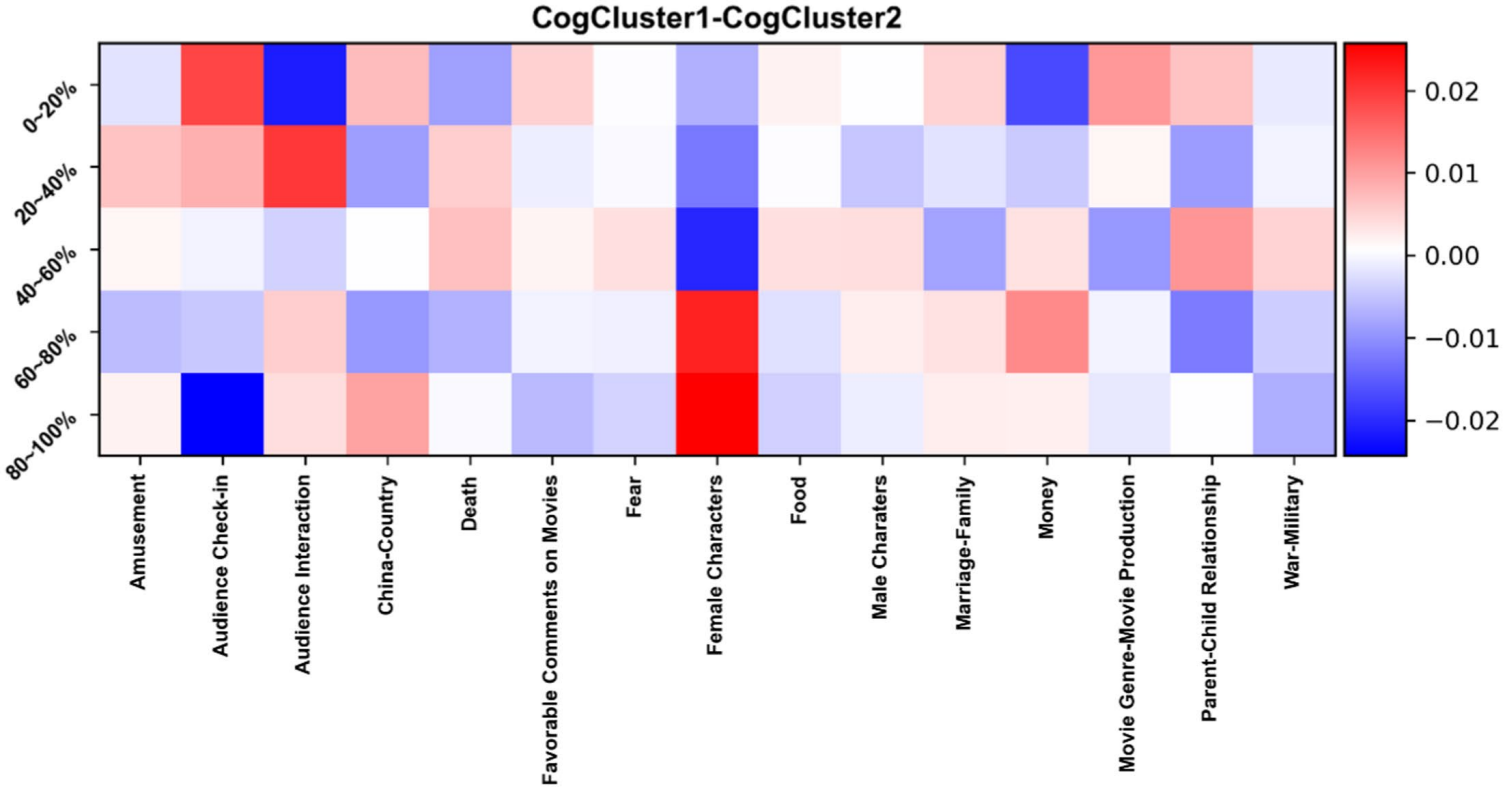
Contrastive heatmap for *CogCluster1* and *CogCluster2*, with differences of prevalence of comments across time slices and topics. A dark red cell means that the proportion of comments is much higher in *CogCluster1* than in *CogCluster2*. A dark blue cell corresponds to the opposite situation.

**Figure 10:**
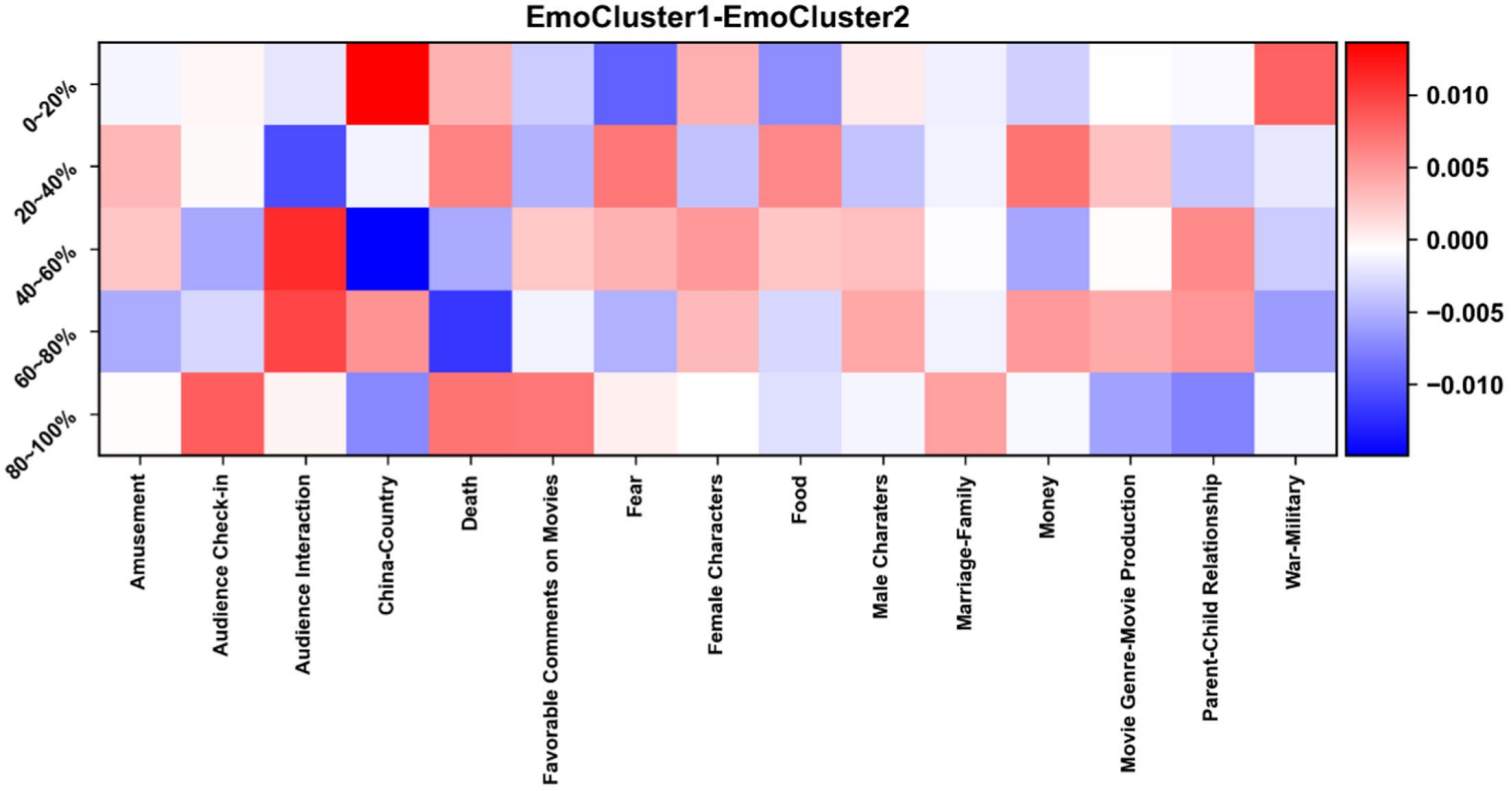
Contrastive heatmap for *EmoCluster1* and *EmoCluster2*, with differences of prevalence of comments across time slices and topics. A dark red cell means that the proportion of comments is much higher in *EmoCluster1* than in *EmoCluster2*. A dark blue cell corresponds to the opposite situation.

To assess this hypothesis, Figures 9, 10 display two contrastive heatmaps, for cognitive complexity and emotionality, respectively. One can observe how the different engagement trajectories – and their respective sets of movies – differ in terms of when and how much the different topics are discussed by viewers.

Regarding cognitive complexity, one clear observation is that **female characters** are more discussed at the end of the movie in ***CogCluster1*** than in ***CogCluster2***. In contrast**, male characters** attract a similar amount of attention across the different time periods in both CogClusters. Also, **check-ins** are more common at the end in ***CogCluster2***, while they are more common at the beginning of a movie in ***CogCluster1***. **Audience interaction** is stronger in the first 20% of a movie in ***CogCluster2***, while the opposite is true for the next time slice. As for emotionality, **China** as a topic is more present in ***EmoCluster1*** than in ***EmoCluster2*** at the beginning of a movie, while it is the opposite around the middle of it. **War-Military** is also stronger in ***EmoCluster1*** at the beginning of a movie. There are many other differences of smaller magnitude, and all in all the differences remain very limited – a difference of 0.01 for instance means a 1%-difference between the by-cluster average percentages of comments for a time slice and a topic.

Given the cognitive complexity and emotionality of the different topics, could the previous differences explain that ***CogCluster1*** and ***EmoCluster1*** correspond to increasing engagement, and ***CogCluster2*** and ***EmoCluster2*** to decreasing engagement? More **interactions** with others at the beginning of the movies in ***CogCluster2*** might align with the different trajectories of the two clusters. So do more **check-ins** at the end of the movies in the same cluster, more “**death**” comments and **favorable comments on movies** at the end of the movies in ***EmoCluster1***, and more **“fear”** comments at the beginnings of the movies in ***EmoCluster2*.** On the other hand, more **“war-military**” comments in ***EmoCluster1*** at the beginning of the movies is rather contradictory.

In brief, observations are not conclusive, effect sizes are very small, and topics thus do not fare better than movie genres to explain engagement trajectories.

#### Relationship between trajectories of screenplays and trajectories of comments

Our previous attempts at accounting for engagement trajectories have not revealed striking patterns. A possible explanation might be that considering movie genres or topics implies averaging across various sets of movies, which blur the processes at work. Considering movies independently from each other might be more meaningful.

When trying to account for the discovered patterns of engagement, it is reasonable to assume they derive at least partly from movie narratives. For instance, when characters are confronted with dilemmas and face cognitive and emotional challenges, a deeply engaged audience may experience similar reactions.

**Figure 11:**
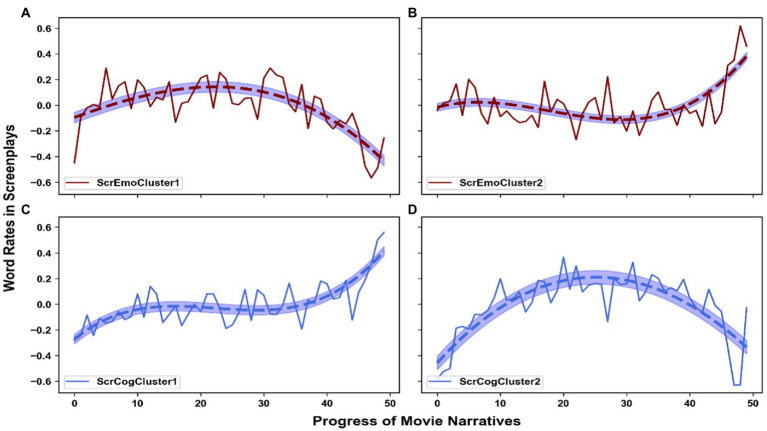
Aggregate time series, i.e., prototypical trajectories, of evolving emotion **(A,B)** and cognition-related **(C,D)** word rates, as generated with soft-DTW k-means clustering. The *X*-axis represents 50 time-slices of screenplay narrative (each corresponds to 2% of the content of the screenplays). The *Y*-axis shows the standardized proportion of emotion-related (upper panels) or cognition-related (lower panels) words used in the screenplays.

The analysis of the screenplays of nearly three quarters of our movies reveals a similar pattern as for comments: two main trajectories can be identified for both emotional content and cognitive content (i.e., emotion and cognition-related word rates calculated by LIWC through time), as presented in Figure 11. We call the four clusters **ScrEmoCluster1** (90 movies), **ScrEmoCluster2** (89 movies), **ScrCogCluster1** (106 movies), and **ScrCogCluster2** (73 movies).

Only the trajectory of ***ScrCogCluster1*** is monotonic (*p* < 0.05), with an increasing trend. ***ScrEmoCluster1*** and ***ScrCogCluster2*** display a reversed U-shape, while ***ScrEmoCluster2*** presents a more saddle-like shape. As a first macro-level observation, movie emotional and cognitive content does not seem to closely match viewers’ engagement. Chi^2^-tests also revealed no significant association between comments and screenplays, either for cognitive clusters or emotional clusters. The situation can be, however, further analyzed at the level of each movie.

**Figure 12:**
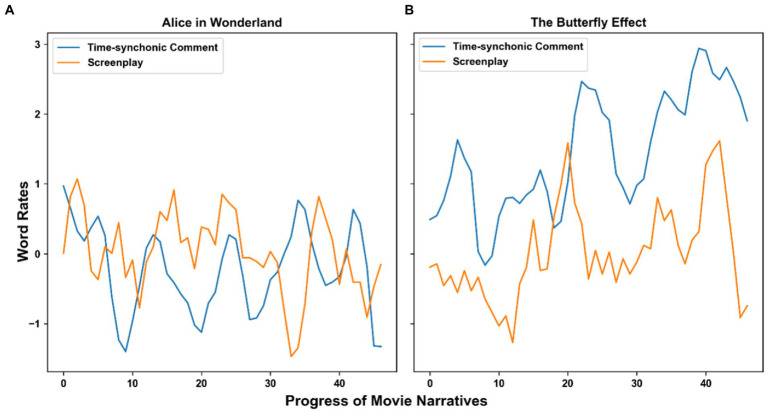
Trajectories of cognitive content in the screenplay and of viewers’ cognitive engagement in two contrasting situations: **(A)** the movie *Alice in Wonderland*, for which there is no Granger-causality and **(B)** the movie *The Butterfly Effect*, for which the cognitive content detected in the screenplay Granger-causes the audience’s cognitive response. For visualization purposes, each time series was smoothed with a moving average with a window size of 3. The Granger-causality test was done on the time series without smoothing.

Only 26 among 178 movie screenplays Granger-cause the cognitive activity of the audience (with a significance level of 0.05) with a lag of 5 or less. Only 23 screenplays Granger-cause the emotional trajectory of the audience (with the same significance level and maximum lag). The names of these movies can be found in a table in the Supplementary Material S7. Figure 12 reports two examples: the movie “*The Butterfly Effect*,” for which there is Granger causality, and the movie “*Alice in Wonderland*,” for which there is not. In the former case, the two trajectories show a similar variation trend, with the one of the screenplay preceding the one of the comments, though not all corresponding peaks and valleys have the exact same shape and lag.

We could not find reasonable explanations for why the narrative content of some movies seemed to partly predict their audiences’ cognitive or emotional engagement through time, and why other movies “behaved” differently.

To summarize, there seem to be few connections between the unfolding of narrative content and the emotional and cognitive nature of comments through movie time.

## Discussion

We have considered a range of successive investigations to understand an audience’s reaction to the unfolding narratives of a large number of movies belonging to different genres. Our results raise questions, in particular at the intersection between movie content and viewers’ reactions.

### Emotion and cognition in unfolding movie narrative

We found two prototypical trajectories for both emotional content and cognitive content in movie screenplays analyzed with SCLIWC. Such large-scale fluctuations of cognition and emotion through time in movies are reminiscent of Reagan et al. (2016) emotional arcs and Boyd et al. (2020) narrative arcs, which reveal core structures of emotional experience and narratives in literary work, respectively.

One can wonder why we did not identify the six “basic shapes” reported by Reagan et al. (2016). Different explanations might be suggested, from a different size of corpus (178 screenplays versus 1,327 novels) to the different extraction techniques of the prototypical trajectories (soft-DTW k-means clustering versus singular value decomposition), to the difference between novels and screenplays – the later focusing on character’s speech and lacking the rich inner and outer descriptions found in many novels. The sensitivity of the clustering of time series to the clustering technique used is a current limitation of our work, and can be further studied by comparing various algorithms. Such algorithms do not yield a “correct answer” but instead offer a view on the data which must be considered as such.

The reversed u-shape trajectory of cognition-related word rates for some of the movies (***ScrCogCluster2***) corresponds to the shape of the so-called cognitive tension curve in novel narratives discussed by Boyd et al. (2020) (this curve was also generated by calculating cognition-related word rates with LIWC on a series of text segments). However, our second cluster, ***ScrCogCluster1*** (106 out of 179 movies), displays an upward trend with two periods of increase at the beginning and at the end of the movie, separated by a period of near stability. What essentially differs between the two clusters we found is the upward or downward evolution in the final part of the movie. This raises the question of whether the resolution of a given plot may differ at the surface level when exposed in a novel or in a screenplay.

### Disconnection between narrative content and viewers’ reactions

A reasonable *a-priori* expectation at the onset of our study was the existence of a relationship between movie content and viewers’ reactions. Of course, a movie is a complex of genre, screenplay, audio, shooting, lighting, and mise-en-scene, but it made sense to predict a clear impact of the first two elements. We found, however, that the connections were tenuous at best: movie genres impact on linguistic and psychological dimensions, topics, and audience cognitive and emotional engagement, but only to some extent, with small effect sizes for the significant effects. Narratives, despite being finer-grained than genres to describe movies, do not appear to exert much influence either on viewers’ engagement trajectories. While, as explained above, this may be due to the quantitative techniques we relied upon, other explanations can be suggested.

First, at a micro-level, while the challenges faced by movie protagonists may lead viewers to experience what these characters experience, a different perspective on the unfolding events might easily occur. Suffering on screen may for instance, be met with sympathy, indifference or amusement, depending on the movie genre (e.g., a tragicomedy for the last reaction). Conversely, characters unaware of or enduring their misfortune may create sadness or anxiety in viewers. Viewers may analyze and predict the situation and subplot more deeply than the protagonists etc. A number of possibilities exist, which may translate to the macro-level. Additionally, one may wonder whether viewers who are truly engaged with the movie spend time to input comments. Would there be a difference between them and viewers who comment?

Second, concentrating on screenplays, and therefore protagonists’ speech, masks movie soundtracks and staging, which also contribute to viewers’ reactions and engagement (Hoeckner et al., 2011; Tan, 2018; Steffens, 2020).

Third, viewers do not only comment on movie content but also on other viewers’ comments, which are not necessarily directly tied to the screenplay. Recurrent interactions can generate crowd “herd effects” in the commenting behavior (He et al., 2017), which may, at least partly, underlie the shape of engagement trajectories. This has been left untouched in our study. Trying to detect relatedness between messages and unrelatedness to the movie content – not an easy task – could help to trim comments to focus on the most relevant ones with respect to movie content.

We found an interesting effect of movie age on audience cognitive engagement, with older movies promoting increasingly deep analytical behavior. We can offer two explanations to this effect: (i) the audience’s respect toward older and more classic movies (for instance, *Blade Runner*, which was released in 1982), translating to a progressively deeper analytical engagement to ponder over the movie’s plot and making or (ii) an evolution of storytelling in movies, with older movies offering increasingly manipulative and thought-provoking plots.

### Social and cultural setting of the study

One needs to be aware that the social and cultural background of the viewers in our study likely have some influence on our results.

In socio-economical terms, Tencent Video is a paid server, which means that commenting viewers are unlikely to faithfully represent the whole population: they have the financial capacity to pay for the service, a good-enough internet connection, an interest in leaving comments, and good literacy. Assuming that these elements translate to a certain social and educational level, the audience is more likely to be classified as working class, with a medium level of education or above. A previous study has revealed that audience demographics (gender, age range, education, social class, etc.) are associated with different movie features (for instance, country of origin, genre, and promotional effort; Redondo and Holbrook, 2010). The authors found that audiences with the highest level of education and social class are more willing to watch movies that are critical and lack sentimentality. In contrast, lower social class moviegoers choose mainly emotionally provoking movies. In our case, audience engagement’s descending and ascending trajectories could perhaps be a product of demographic segmentations too. However, as demographic features of the senders of time-synchronic comment are unavailable, we were limited in our investigations.

Secondly, all the comments came from a Chinese audience. This means that they likely reflect some cultural factors, something suggested quite straightforwardly by our topic analysis when it highlights a topic centered on China and its connection with other countries. This topic appears whatever the movie genre and even if all our movies but one were not made in China nor had Chinese cultural references. Other topics may also be colored by the origins of the viewers, though less obviously.

A possible cultural gap might thus exist between movie content and viewers’ reaction to it. Whether this explains the disconnection outlined previously is an open question. Earlier research has revealed the gap between Chinese and US movies in terms of satisfaction of curiosity, moral education, and plot (Yang and Liu, 2017). For instance, the Chinese audience values US movies as more logical, fascinating, and unexpected than Chinese movies, for Chinese movies are more likely to express “main melody” political values, which is less attractive (*ibid*). For that reason, would a Chinese audience’s commenting behavior change drastically with Chinese movies, with a stronger sense of aesthetic fatigue due to large proportions of sociopolitical elements? Would the enjoyment differ to the point that different engagement patterns would emerge? Obviously, time-synchronic comments in other languages and from a range of different cultural groups would help address these questions. They remain to this day, however, a very East-Asian phenomenon.

The regular mentioning of “China,” or comparisons of China with other countries made by the audience suggests that movies with a foreign setting may trigger a sense of group belonging or of collective identity (Brewer and Gardner, 1996) when the audience is experiencing the movie together, especially when the movie is political, technological, or social, and even if is not a patriotic one made by Chinese producers. To examine this further, we examined the proportion of the word “we” among topics, as it can be indicative of a shared identity (Simmons et al., 2005; Luo et al., 2020). We found that among the topics, the proportion was highest when people talk about China or its relationship with other countries (0.57%, to be compared to 0.18% – the average word rate of “we” in topics excluding “China”).

### Time-synchronic comments and crowd behavior

Our study hopefully reveals time-synchronic comment’s potential as a cross-disciplinary research object, with reference especially to digital humanities where advanced quantitative techniques intersect with questions on human nature and psychology. In this light, time-synchronic and, more broadly, live streaming comments open a window on the crowd mentality taking place when people face the same event together, as shown by some studies. In particular, Luo et al. (2020) found an amplification effect when comparing emotions extracted from YouTube live streaming comments on the news to emotions of static retrospective comments. Watching and commenting on the video *together* increase emotions compared to a situation where others’ comments are available but do not come from a shared simultaneous experience. Knowing that others face an event right as we do too may drive our emotions up, pointing at our inherently social nature and its psychological consequences. One may refer here to the theory of “shared attention” (Shteynberg et al., 2014; Shteynberg, 2015).

Additionally, when analyzing how the intensity of emotions changes through time, Luo et al. (2020) found a single monotonous upward trend. This corresponds to only one of our two trajectories of emotional engagement. This monotony might derive from the nature of the videos – news versus movies –, and points at the need to consider various contents and their comments to appreciate the range of possible reactions from the crowd.

## Conclusion

Our study explored an emerging source of social media data, time-synchronic “Danmu” comments, to reveal an audience’s psychological reaction when watching movies. On the basis of more than 3,000,000 comments related to 240 well-known movies, we found that:

1. Two key dimensions of viewers’ commenting behaviors lie in the formality of their language and their mental states. The latter further consists of emotions and cognitive/analytical reasoning;
2. A number of topics can be identified and studied through (movie) time. These topics reflect at least partly the viewers’ sociocultural background;
3. Audience engagement can be decomposed into emotional and cognitive engagement throughout a movie. For each of them, two prototypical broad-level trajectories exist: an upward trend and a downward trend;
4. These trajectories are only little determined by movie content (cognitive and emotional load of the screenplay, movie genre) or by topics. This may be explained by differences of perspective between movie characters and movie viewers, or the interactive nature of the comments.

Despite some limitations, these results highlight the uniqueness and value of time-synchronic comments as a dynamic natural language resource that can give insights into how people react to a narrative process and its unfolding events and, more broadly, how a crowd reacts to a shared event.

## Data availability statement

The raw data supporting the conclusions of this article will be made available by the authors, without undue reservation.

## Author contributions

WN contributed to the data collection and design of the study, organized the database, performed the statistical analysis, and wrote the first draft of the manuscript. CC contributed to the design of the study, supervised the study, made revisions during the analysis process, wrote sections of the manuscript, and reorganized and polished the manuscript. All authors contributed to manuscript revision, read, and approved the submitted version.

## Conflict of interest

The authors declare that the research was conducted in the absence of any commercial or financial relationships that could be construed as a potential conflict of interest.

## Publisher’s note

All claims expressed in this article are solely those of the authors and do not necessarily represent those of their affiliated organizations, or those of the publisher, the editors and the reviewers. Any product that may be evaluated in this article, or claim that may be made by its manufacturer, is not guaranteed or endorsed by the publisher.

## Footnotes

^1^ https://www.huajiao.com/

^2^ https://v.qq.com

^3^ https://github.com/wenjingni/Time-synchronic-comments-analysis

^4^ https://www.imdb.com/feature/genre/
