# Supplementary figures and images for: Time-synchronic comments on video streaming website reveal core structures of audience engagement in movie viewing
Source: Front Psychol. 2023 Jan 19;13:1040755. doi: 10.3389/fpsyg.2022.1040755 (PMC9893864; doi:10.3389/fpsyg.2022.1040755)

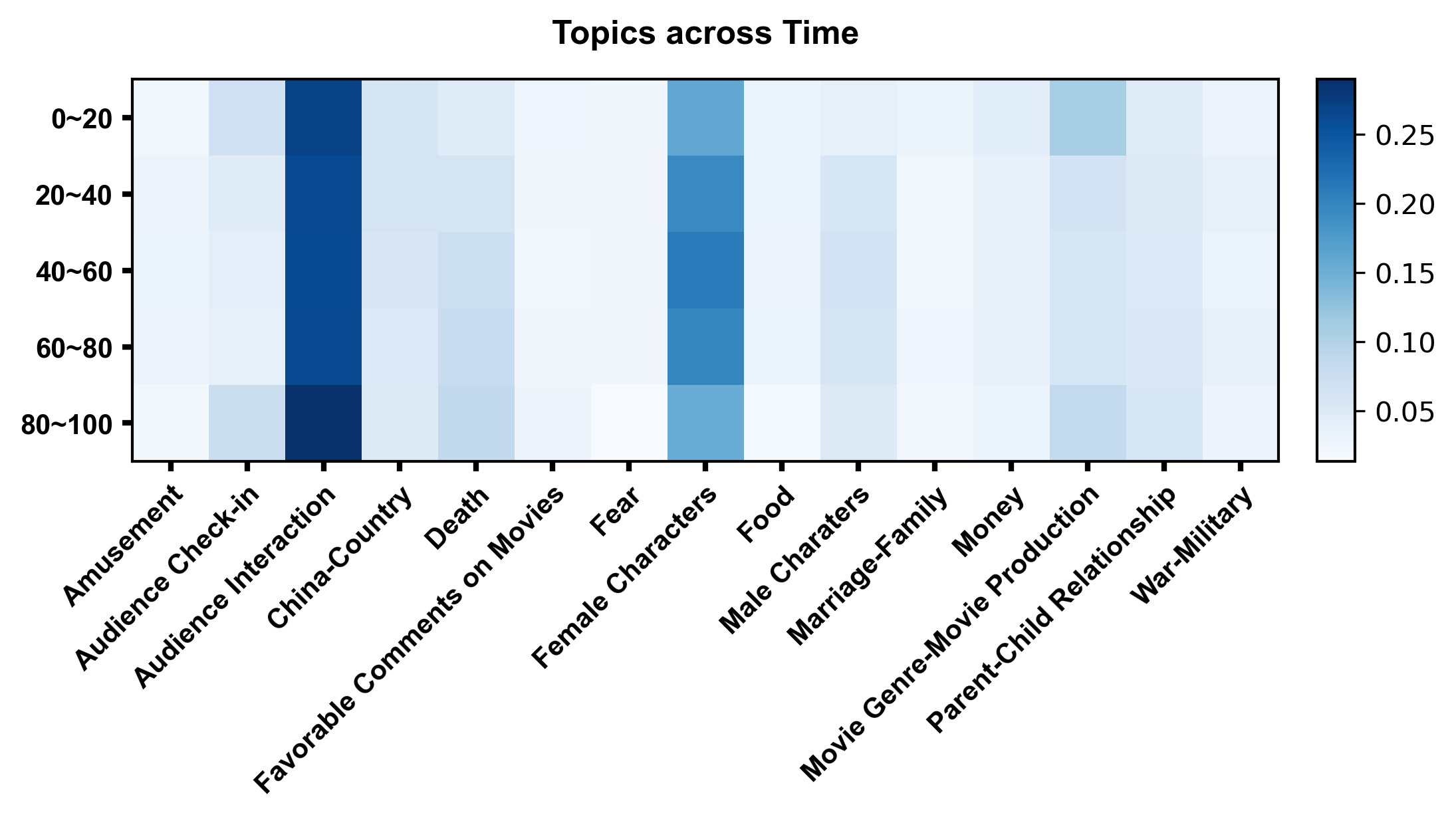

Supplement: Supplementary file 4 [file Image_1.TIFF]

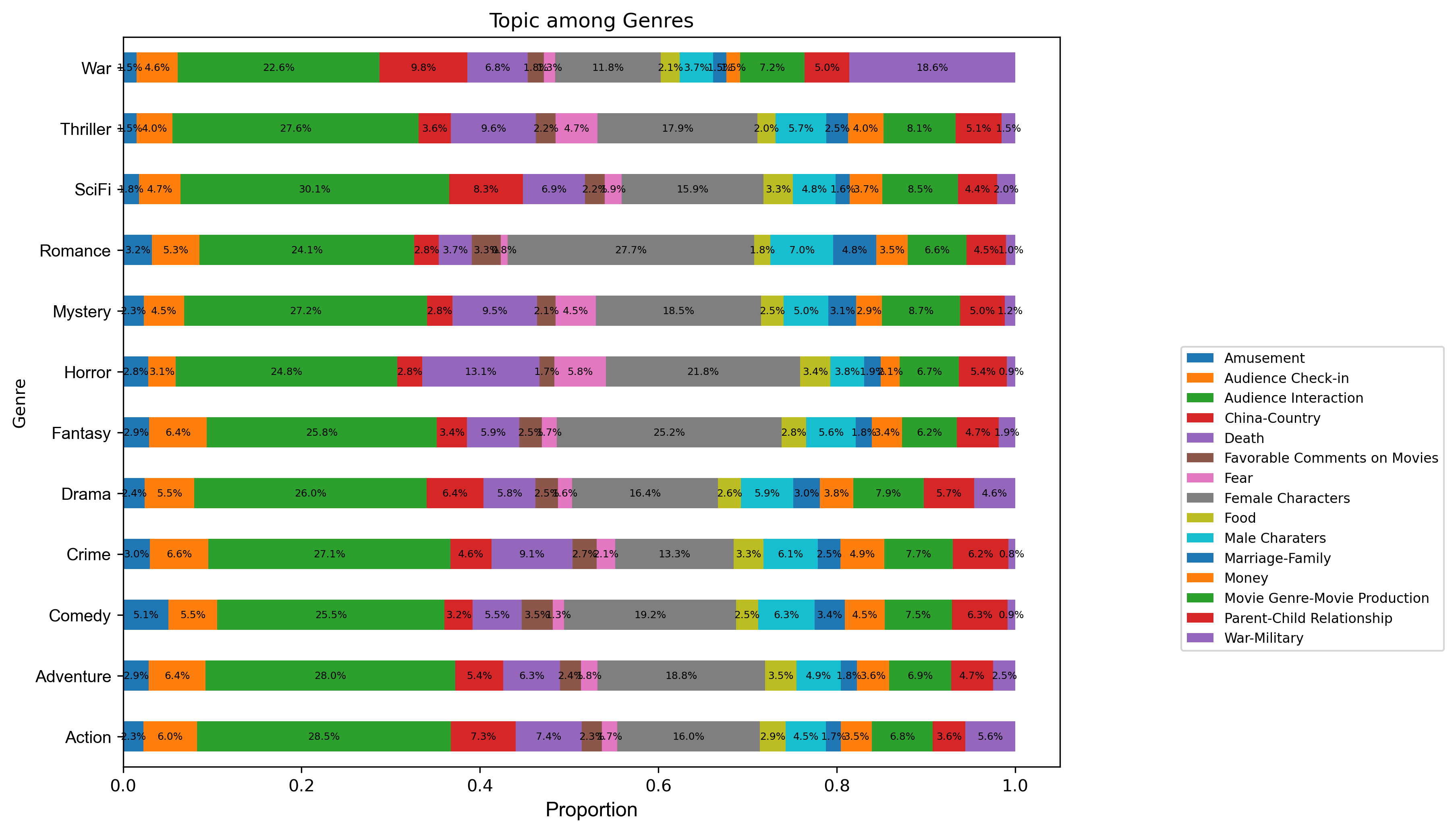

Supplement: Supplementary file 5 [file Image_2.TIFF]
