# Supplementary material for: Time-synchronic comments on video streaming website reveal core structures of audience engagement in movie viewing
Source: Front Psychol. 2023 Jan 19;13:1040755. doi: 10.3389/fpsyg.2022.1040755 (PMC9893864; doi:10.3389/fpsyg.2022.1040755)
Supplement: Supplementary file 6 [file Table_1.DOC]

Supplementary Material 1:
Tukey's post-hoc test for MANOVA

Multiple Comparisons	
Tukey HSD  	
Dependent Variable	(I) Genre	(J) Genre	Mean Difference (I-J)	Std. Error	Sig.	95% Confidence Interval	
						Lower Bound	Upper Bound	
Linguistic	Actio	Adven	-.0007	.01287	1.000	-.0429	.0415	
		Comed	-.0682*	.01408	.000	-.1144	-.0220	
		Crime	-.0251	.01567	.908	-.0765	.0263	
		Drama	-.0588*	.01078	.000	-.0942	-.0234	
		Fanta	-.0342	.01464	.451	-.0822	.0138	
		Horro	-.0540*	.01625	.044	-.1073	-.0007	
		Myste	-.0545*	.01339	.003	-.0985	-.0106	
		Roman	-.0982*	.01389	.000	-.1438	-.0527	
		SciFi	-.0440*	.01305	.038	-.0868	-.0012	
		Thril	-.0532*	.01347	.005	-.0973	-.0090	
		War	.0035	.01669	1.000	-.0512	.0583	
	Adven	Actio	.0007	.01287	1.000	-.0415	.0429	
		Comed	-.0675*	.01428	.000	-.1143	-.0206	
		Crime	-.0244	.01585	.928	-.0764	.0276	
		Drama	-.0581*	.01104	.000	-.0943	-.0219	
		Fanta	-.0335	.01482	.505	-.0822	.0151	
		Horro	-.0533	.01642	.056	-.1071	.0006	
		Myste	-.0539*	.01360	.005	-.0985	-.0092	
		Roman	-.0976*	.01409	.000	-.1438	-.0513	
		SciFi	-.0433	.01326	.052	-.0868	.0002	
		Thril	-.0525*	.01367	.008	-.0973	-.0076	
		War	.0042	.01686	1.000	-.0511	.0595	
	Comed	Actio	.0682*	.01408	.000	.0220	.1144	
		Adven	.0675*	.01428	.000	.0206	.1143	
		Crime	.0431	.01684	.307	-.0122	.0983	
		Drama	.0094	.01243	1.000	-.0314	.0501	
		Fanta	.0339	.01589	.596	-.0182	.0861	
		Horro	.0142	.01738	1.000	-.0428	.0712	
		Myste	.0136	.01475	.999	-.0348	.0620	
		Roman	-.0301	.01520	.708	-.0799	.0198	
		SciFi	.0242	.01444	.879	-.0232	.0715	
		Thril	.0150	.01482	.997	-.0336	.0636	
		War	.0717*	.01780	.004	.0133	.1301	
	Crime	Actio	.0251	.01567	.908	-.0263	.0765	
		Adven	.0244	.01585	.928	-.0276	.0764	
		Comed	-.0431	.01684	.307	-.0983	.0122	
		Drama	-.0337	.01420	.426	-.0803	.0129	
		Fanta	-.0091	.01731	1.000	-.0659	.0477	
		Horro	-.0289	.01869	.927	-.0902	.0325	
		Myste	-.0294	.01627	.813	-.0828	.0239	
		Roman	-.0731*	.01668	.001	-.1279	-.0184	
		SciFi	-.0189	.01599	.990	-.0714	.0336	
		Thril	-.0281	.01633	.860	-.0816	.0255	
		War	.0286	.01908	.940	-.0340	.0912	
	Drama	Actio	.0588*	.01078	.000	.0234	.0942	
		Adven	.0581*	.01104	.000	.0219	.0943	
		Comed	-.0094	.01243	1.000	-.0501	.0314	
		Crime	.0337	.01420	.426	-.0129	.0803	
		Fanta	.0246	.01305	.770	-.0183	.0674	
		Horro	.0048	.01484	1.000	-.0438	.0535	
		Myste	.0043	.01164	1.000	-.0339	.0425	
		Roman	-.0394	.01221	.058	-.0795	.0006	
		SciFi	.0148	.01125	.977	-.0221	.0517	
		Thril	.0056	.01173	1.000	-.0328	.0441	
		War	.0623*	.01532	.003	.0120	.1126	
	Fanta	Actio	.0342	.01464	.451	-.0138	.0822	
		Adven	.0335	.01482	.505	-.0151	.0822	
		Comed	-.0339	.01589	.596	-.0861	.0182	
		Crime	.0091	.01731	1.000	-.0477	.0659	
		Drama	-.0246	.01305	.770	-.0674	.0183	
		Horro	-.0197	.01783	.994	-.0783	.0388	
		Myste	-.0203	.01528	.975	-.0705	.0298	
		Roman	-.0640*	.01572	.003	-.1156	-.0125	
		SciFi	-.0098	.01498	1.000	-.0589	.0394	
		Thril	-.0189	.01535	.986	-.0693	.0314	
		War	.0377	.01824	.645	-.0221	.0976	
	Horro	Actio	.0540*	.01625	.044	.0007	.1073	
		Adven	.0533	.01642	.056	-.0006	.1071	
		Comed	-.0142	.01738	1.000	-.0712	.0428	
		Crime	.0289	.01869	.927	-.0325	.0902	
		Drama	-.0048	.01484	1.000	-.0535	.0438	
		Fanta	.0197	.01783	.994	-.0388	.0783	
		Myste	-.0006	.01683	1.000	-.0558	.0546	
		Roman	-.0443	.01722	.299	-.1008	.0122	
		SciFi	.0100	.01656	1.000	-.0443	.0643	
		Thril	.0008	.01689	1.000	-.0546	.0562	
		War	.0575	.01956	.130	-.0067	.1216	
	Myste	Actio	.0545*	.01339	.003	.0106	.0985	
		Adven	.0539*	.01360	.005	.0092	.0985	
		Comed	-.0136	.01475	.999	-.0620	.0348	
		Crime	.0294	.01627	.813	-.0239	.0828	
		Drama	-.0043	.01164	1.000	-.0425	.0339	
		Fanta	.0203	.01528	.975	-.0298	.0705	
		Horro	.0006	.01683	1.000	-.0546	.0558	
		Roman	-.0437	.01456	.111	-.0915	.0041	
		SciFi	.0106	.01377	1.000	-.0346	.0557	
		Thril	.0014	.01416	1.000	-.0451	.0479	
		War	.0581*	.01726	.039	.0014	.1147	
	Roman	Actio	.0982*	.01389	.000	.0527	.1438	
		Adven	.0976*	.01409	.000	.0513	.1438	
		Comed	.0301	.01520	.708	-.0198	.0799	
		Crime	.0731*	.01668	.001	.0184	.1279	
		Drama	.0394	.01221	.058	-.0006	.0795	
		Fanta	.0640*	.01572	.003	.0125	.1156	
		Horro	.0443	.01722	.299	-.0122	.1008	
		Myste	.0437	.01456	.111	-.0041	.0915	
		SciFi	.0542*	.01425	.008	.0075	.1010	
		Thril	.0451	.01463	.089	-.0029	.0931	
		War	.1018*	.01765	.000	.0439	.1597	
	SciFi	Actio	.0440*	.01305	.038	.0012	.0868	
		Adven	.0433	.01326	.052	-.0002	.0868	
		Comed	-.0242	.01444	.879	-.0715	.0232	
		Crime	.0189	.01599	.990	-.0336	.0714	
		Drama	-.0148	.01125	.977	-.0517	.0221	
		Fanta	.0098	.01498	1.000	-.0394	.0589	
		Horro	-.0100	.01656	1.000	-.0643	.0443	
		Myste	-.0106	.01377	1.000	-.0557	.0346	
		Roman	-.0542*	.01425	.008	-.1010	-.0075	
		Thril	-.0092	.01384	1.000	-.0546	.0362	
		War	.0475	.01700	.184	-.0083	.1033	
	Thril	Actio	.0532*	.01347	.005	.0090	.0973	
		Adven	.0525*	.01367	.008	.0076	.0973	
		Comed	-.0150	.01482	.997	-.0636	.0336	
		Crime	.0281	.01633	.860	-.0255	.0816	
		Drama	-.0056	.01173	1.000	-.0441	.0328	
		Fanta	.0189	.01535	.986	-.0314	.0693	
		Horro	-.0008	.01689	1.000	-.0562	.0546	
		Myste	-.0014	.01416	1.000	-.0479	.0451	
		Roman	-.0451	.01463	.089	-.0931	.0029	
		SciFi	.0092	.01384	1.000	-.0362	.0546	
		War	.0567	.01732	.051	-.0001	.1135	
	War	Actio	-.0035	.01669	1.000	-.0583	.0512	
		Adven	-.0042	.01686	1.000	-.0595	.0511	
		Comed	-.0717*	.01780	.004	-.1301	-.0133	
		Crime	-.0286	.01908	.940	-.0912	.0340	
		Drama	-.0623*	.01532	.003	-.1126	-.0120	
		Fanta	-.0377	.01824	.645	-.0976	.0221	
		Horro	-.0575	.01956	.130	-.1216	.0067	
		Myste	-.0581*	.01726	.039	-.1147	-.0014	
		Roman	-.1018*	.01765	.000	-.1597	-.0439	
		SciFi	-.0475	.01700	.184	-.1033	.0083	
		Thril	-.0567	.01732	.051	-.1135	.0001	
Psycho	Actio	Adven	-.0010	.01022	1.000	-.0345	.0326	
		Comed	-.0591*	.01119	.000	-.0958	-.0224	
		Crime	-.0139	.01245	.994	-.0547	.0269	
		Drama	-.0648*	.00857	.000	-.0929	-.0367	
		Fanta	-.0219	.01163	.771	-.0600	.0163	
		Horro	-.0328	.01291	.318	-.0751	.0096	
		Myste	-.0323	.01064	.101	-.0672	.0026	
		Roman	-.1005*	.01103	.000	-.1367	-.0643	
		SciFi	-.0285	.01037	.204	-.0626	.0055	
		Thril	-.0345	.01070	.059	-.0696	.0006	
		War	-.0550*	.01326	.002	-.0985	-.0115	
	Adven	Actio	.0010	.01022	1.000	-.0326	.0345	
		Comed	-.0581*	.01134	.000	-.0953	-.0209	
		Crime	-.0129	.01259	.997	-.0542	.0284	
		Drama	-.0638*	.00877	.000	-.0926	-.0350	
		Fanta	-.0209	.01178	.832	-.0595	.0178	
		Horro	-.0318	.01304	.382	-.0746	.0110	
		Myste	-.0313	.01080	.144	-.0667	.0041	
		Roman	-.0996*	.01119	.000	-.1363	-.0628	
		SciFi	-.0276	.01053	.273	-.0621	.0070	
		Thril	-.0336	.01086	.087	-.0692	.0021	
		War	-.0540*	.01339	.004	-.0979	-.0101	
	Comed	Actio	.0591*	.01119	.000	.0224	.0958	
		Adven	.0581*	.01134	.000	.0209	.0953	
		Crime	.0452*	.01338	.037	.0013	.0891	
		Drama	-.0057	.00987	1.000	-.0381	.0267	
		Fanta	.0372	.01262	.127	-.0042	.0786	
		Horro	.0263	.01381	.755	-.0190	.0716	
		Myste	.0268	.01172	.487	-.0116	.0652	
		Roman	-.0414*	.01207	.031	-.0811	-.0018	
		SciFi	.0305	.01147	.247	-.0071	.0682	
		Thril	.0245	.01177	.634	-.0141	.0632	
		War	.0041	.01414	1.000	-.0423	.0505	
	Crime	Actio	.0139	.01245	.994	-.0269	.0547	
		Adven	.0129	.01259	.997	-.0284	.0542	
		Comed	-.0452*	.01338	.037	-.0891	-.0013	
		Drama	-.0509*	.01128	.000	-.0879	-.0139	
		Fanta	-.0080	.01375	1.000	-.0531	.0372	
		Horro	-.0189	.01485	.982	-.0676	.0298	
		Myste	-.0184	.01293	.959	-.0608	.0240	
		Roman	-.0866*	.01325	.000	-.1301	-.0431	
		SciFi	-.0146	.01270	.992	-.0563	.0270	
		Thril	-.0206	.01298	.912	-.0632	.0219	
		War	-.0411	.01516	.224	-.0908	.0086	
	Drama	Actio	.0648*	.00857	.000	.0367	.0929	
		Adven	.0638*	.00877	.000	.0350	.0926	
		Comed	.0057	.00987	1.000	-.0267	.0381	
		Crime	.0509*	.01128	.000	.0139	.0879	
		Fanta	.0429*	.01037	.002	.0089	.0769	
		Horro	.0320	.01179	.220	-.0066	.0707	
		Myste	.0325*	.00925	.024	.0022	.0628	
		Roman	-.0357*	.00970	.013	-.0676	-.0039	
		SciFi	.0362*	.00893	.003	.0069	.0656	
		Thril	.0302	.00932	.056	-.0003	.0608	
		War	.0098	.01217	1.000	-.0301	.0497	
	Fanta	Actio	.0219	.01163	.771	-.0163	.0600	
		Adven	.0209	.01178	.832	-.0178	.0595	
		Comed	-.0372	.01262	.127	-.0786	.0042	
		Crime	.0080	.01375	1.000	-.0372	.0531	
		Drama	-.0429*	.01037	.002	-.0769	-.0089	
		Horro	-.0109	.01417	1.000	-.0574	.0356	
		Myste	-.0104	.01214	.999	-.0502	.0294	
		Roman	-.0787*	.01248	.000	-.1196	-.0377	
		SciFi	-.0067	.01190	1.000	-.0457	.0324	
		Thril	-.0127	.01219	.997	-.0527	.0273	
		War	-.0331	.01449	.488	-.0807	.0144	
	Horro	Actio	.0328	.01291	.318	-.0096	.0751	
		Adven	.0318	.01304	.382	-.0110	.0746	
		Comed	-.0263	.01381	.755	-.0716	.0190	
		Crime	.0189	.01485	.982	-.0298	.0676	
		Drama	-.0320	.01179	.220	-.0707	.0066	
		Fanta	.0109	.01417	1.000	-.0356	.0574	
		Myste	.0005	.01337	1.000	-.0434	.0443	
		Roman	-.0678*	.01368	.000	-.1127	-.0229	
		SciFi	.0042	.01315	1.000	-.0389	.0474	
		Thril	-.0018	.01342	1.000	-.0458	.0422	
		War	-.0222	.01554	.957	-.0732	.0288	
	Myste	Actio	.0323	.01064	.101	-.0026	.0672	
		Adven	.0313	.01080	.144	-.0041	.0667	
		Comed	-.0268	.01172	.487	-.0652	.0116	
		Crime	.0184	.01293	.959	-.0240	.0608	
		Drama	-.0325*	.00925	.024	-.0628	-.0022	
		Fanta	.0104	.01214	.999	-.0294	.0502	
		Horro	-.0005	.01337	1.000	-.0443	.0434	
		Roman	-.0682*	.01157	.000	-.1062	-.0303	
		SciFi	.0037	.01094	1.000	-.0321	.0396	
		Thril	-.0023	.01125	1.000	-.0392	.0347	
		War	-.0227	.01371	.887	-.0677	.0223	
	Roman	Actio	.1005*	.01103	.000	.0643	.1367	
		Adven	.0996*	.01119	.000	.0628	.1363	
		Comed	.0414*	.01207	.031	.0018	.0811	
		Crime	.0866*	.01325	.000	.0431	.1301	
		Drama	.0357*	.00970	.013	.0039	.0676	
		Fanta	.0787*	.01248	.000	.0377	.1196	
		Horro	.0678*	.01368	.000	.0229	.1127	
		Myste	.0682*	.01157	.000	.0303	.1062	
		SciFi	.0720*	.01132	.000	.0348	.1091	
		Thril	.0660*	.01162	.000	.0278	.1041	
		War	.0455	.01402	.055	-.0004	.0915	
	SciFi	Actio	.0285	.01037	.204	-.0055	.0626	
		Adven	.0276	.01053	.273	-.0070	.0621	
		Comed	-.0305	.01147	.247	-.0682	.0071	
		Crime	.0146	.01270	.992	-.0270	.0563	
		Drama	-.0362*	.00893	.003	-.0656	-.0069	
		Fanta	.0067	.01190	1.000	-.0324	.0457	
		Horro	-.0042	.01315	1.000	-.0474	.0389	
		Myste	-.0037	.01094	1.000	-.0396	.0321	
		Roman	-.0720*	.01132	.000	-.1091	-.0348	
		Thril	-.0060	.01100	1.000	-.0421	.0301	
		War	-.0264	.01350	.721	-.0707	.0179	
	Thril	Actio	.0345	.01070	.059	-.0006	.0696	
		Adven	.0336	.01086	.087	-.0021	.0692	
		Comed	-.0245	.01177	.634	-.0632	.0141	
		Crime	.0206	.01298	.912	-.0219	.0632	
		Drama	-.0302	.00932	.056	-.0608	.0003	
		Fanta	.0127	.01219	.997	-.0273	.0527	
		Horro	.0018	.01342	1.000	-.0422	.0458	
		Myste	.0023	.01125	1.000	-.0347	.0392	
		Roman	-.0660*	.01162	.000	-.1041	-.0278	
		SciFi	.0060	.01100	1.000	-.0301	.0421	
		War	-.0204	.01376	.944	-.0656	.0247	
	War	Actio	.0550*	.01326	.002	.0115	.0985	
		Adven	.0540*	.01339	.004	.0101	.0979	
		Comed	-.0041	.01414	1.000	-.0505	.0423	
		Crime	.0411	.01516	.224	-.0086	.0908	
		Drama	-.0098	.01217	1.000	-.0497	.0301	
		Fanta	.0331	.01449	.488	-.0144	.0807	
		Horro	.0222	.01554	.957	-.0288	.0732	
		Myste	.0227	.01371	.887	-.0223	.0677	
		Roman	-.0455	.01402	.055	-.0915	.0004	
		SciFi	.0264	.01350	.721	-.0179	.0707	
		Thril	.0204	.01376	.944	-.0247	.0656	
Personal	Actio	Adven	.0039	.00255	.928	-.0044	.0123	
		Comed	.0079	.00279	.166	-.0012	.0171	
		Crime	.0026	.00310	1.000	-.0076	.0127	
		Drama	.0049	.00214	.498	-.0022	.0119	
		Fanta	.0059	.00290	.672	-.0036	.0154	
		Horro	.0037	.00322	.993	-.0069	.0142	
		Myste	-.0001	.00265	1.000	-.0088	.0086	
		Roman	.0141*	.00275	.000	.0051	.0232	
		SciFi	.0006	.00258	1.000	-.0079	.0090	
		Thril	-.0007	.00267	1.000	-.0095	.0080	
		War	.0045	.00331	.969	-.0063	.0154	
	Adven	Actio	-.0039	.00255	.928	-.0123	.0044	
		Comed	.0040	.00283	.961	-.0053	.0133	
		Crime	-.0014	.00314	1.000	-.0117	.0089	
		Drama	.0009	.00219	1.000	-.0063	.0081	
		Fanta	.0020	.00294	1.000	-.0077	.0116	
		Horro	-.0003	.00325	1.000	-.0109	.0104	
		Myste	-.0040	.00269	.943	-.0128	.0048	
		Roman	.0102*	.00279	.014	.0011	.0194	
		SciFi	-.0034	.00263	.981	-.0120	.0052	
		Thril	-.0046	.00271	.861	-.0135	.0042	
		War	.0006	.00334	1.000	-.0104	.0115	
	Comed	Actio	-.0079	.00279	.166	-.0171	.0012	
		Adven	-.0040	.00283	.961	-.0133	.0053	
		Crime	-.0054	.00334	.905	-.0163	.0056	
		Drama	-.0031	.00246	.985	-.0111	.0050	
		Fanta	-.0020	.00315	1.000	-.0124	.0083	
		Horro	-.0043	.00344	.986	-.0156	.0070	
		Myste	-.0080	.00292	.209	-.0176	.0016	
		Roman	.0062	.00301	.645	-.0036	.0161	
		SciFi	-.0074	.00286	.297	-.0167	.0020	
		Thril	-.0086	.00293	.129	-.0183	.0010	
		War	-.0034	.00352	.998	-.0150	.0082	
	Crime	Actio	-.0026	.00310	1.000	-.0127	.0076	
		Adven	.0014	.00314	1.000	-.0089	.0117	
		Comed	.0054	.00334	.905	-.0056	.0163	
		Drama	.0023	.00281	1.000	-.0069	.0115	
		Fanta	.0033	.00343	.998	-.0079	.0146	
		Horro	.0011	.00370	1.000	-.0110	.0132	
		Myste	-.0026	.00322	1.000	-.0132	.0079	
		Roman	.0116*	.00330	.024	.0008	.0224	
		SciFi	-.0020	.00317	1.000	-.0124	.0084	
		Thril	-.0033	.00323	.997	-.0139	.0073	
		War	.0020	.00378	1.000	-.0104	.0144	
	Drama	Actio	-.0049	.00214	.498	-.0119	.0022	
		Adven	-.0009	.00219	1.000	-.0081	.0063	
		Comed	.0031	.00246	.985	-.0050	.0111	
		Crime	-.0023	.00281	1.000	-.0115	.0069	
		Fanta	.0010	.00258	1.000	-.0074	.0095	
		Horro	-.0012	.00294	1.000	-.0108	.0084	
		Myste	-.0049	.00231	.594	-.0125	.0026	
		Roman	.0093*	.00242	.007	.0014	.0172	
		SciFi	-.0043	.00223	.743	-.0116	.0030	
		Thril	-.0056	.00232	.411	-.0132	.0021	
		War	-.0003	.00303	1.000	-.0103	.0096	
	Fanta	Actio	-.0059	.00290	.672	-.0154	.0036	
		Adven	-.0020	.00294	1.000	-.0116	.0077	
		Comed	.0020	.00315	1.000	-.0083	.0124	
		Crime	-.0033	.00343	.998	-.0146	.0079	
		Drama	-.0010	.00258	1.000	-.0095	.0074	
		Horro	-.0022	.00353	1.000	-.0138	.0094	
		Myste	-.0060	.00303	.712	-.0159	.0040	
		Roman	.0083	.00311	.252	-.0019	.0185	
		SciFi	-.0053	.00297	.821	-.0151	.0044	
		Thril	-.0066	.00304	.571	-.0166	.0034	
		War	-.0014	.00361	1.000	-.0132	.0105	
	Horro	Actio	-.0037	.00322	.993	-.0142	.0069	
		Adven	.0003	.00325	1.000	-.0104	.0109	
		Comed	.0043	.00344	.986	-.0070	.0156	
		Crime	-.0011	.00370	1.000	-.0132	.0110	
		Drama	.0012	.00294	1.000	-.0084	.0108	
		Fanta	.0022	.00353	1.000	-.0094	.0138	
		Myste	-.0037	.00333	.994	-.0147	.0072	
		Roman	.0105	.00341	.090	-.0007	.0217	
		SciFi	-.0031	.00328	.999	-.0138	.0077	
		Thril	-.0044	.00334	.978	-.0153	.0066	
		War	.0009	.00387	1.000	-.0118	.0136	
	Myste	Actio	.0001	.00265	1.000	-.0086	.0088	
		Adven	.0040	.00269	.943	-.0048	.0128	
		Comed	.0080	.00292	.209	-.0016	.0176	
		Crime	.0026	.00322	1.000	-.0079	.0132	
		Drama	.0049	.00231	.594	-.0026	.0125	
		Fanta	.0060	.00303	.712	-.0040	.0159	
		Horro	.0037	.00333	.994	-.0072	.0147	
		Roman	.0142*	.00288	.000	.0048	.0237	
		SciFi	.0006	.00273	1.000	-.0083	.0096	
		Thril	-.0006	.00280	1.000	-.0098	.0086	
		War	.0046	.00342	.972	-.0066	.0158	
	Roman	Actio	-.0141*	.00275	.000	-.0232	-.0051	
		Adven	-.0102*	.00279	.014	-.0194	-.0011	
		Comed	-.0062	.00301	.645	-.0161	.0036	
		Crime	-.0116*	.00330	.024	-.0224	-.0008	
		Drama	-.0093*	.00242	.007	-.0172	-.0014	
		Fanta	-.0083	.00311	.252	-.0185	.0019	
		Horro	-.0105	.00341	.090	-.0217	.0007	
		Myste	-.0142*	.00288	.000	-.0237	-.0048	
		SciFi	-.0136*	.00282	.000	-.0228	-.0043	
		Thril	-.0149*	.00290	.000	-.0244	-.0054	
		War	-.0096	.00349	.203	-.0211	.0018	
	SciFi	Actio	-.0006	.00258	1.000	-.0090	.0079	
		Adven	.0034	.00263	.981	-.0052	.0120	
		Comed	.0074	.00286	.297	-.0020	.0167	
		Crime	.0020	.00317	1.000	-.0084	.0124	
		Drama	.0043	.00223	.743	-.0030	.0116	
		Fanta	.0053	.00297	.821	-.0044	.0151	
		Horro	.0031	.00328	.999	-.0077	.0138	
		Myste	-.0006	.00273	1.000	-.0096	.0083	
		Roman	.0136*	.00282	.000	.0043	.0228	
		Thril	-.0013	.00274	1.000	-.0103	.0077	
		War	.0040	.00337	.991	-.0071	.0150	
	Thril	Actio	.0007	.00267	1.000	-.0080	.0095	
		Adven	.0046	.00271	.861	-.0042	.0135	
		Comed	.0086	.00293	.129	-.0010	.0183	
		Crime	.0033	.00323	.997	-.0073	.0139	
		Drama	.0056	.00232	.411	-.0021	.0132	
		Fanta	.0066	.00304	.571	-.0034	.0166	
		Horro	.0044	.00334	.978	-.0066	.0153	
		Myste	.0006	.00280	1.000	-.0086	.0098	
		Roman	.0149*	.00290	.000	.0054	.0244	
		SciFi	.0013	.00274	1.000	-.0077	.0103	
		War	.0052	.00343	.933	-.0060	.0165	
	War	Actio	-.0045	.00331	.969	-.0154	.0063	
		Adven	-.0006	.00334	1.000	-.0115	.0104	
		Comed	.0034	.00352	.998	-.0082	.0150	
		Crime	-.0020	.00378	1.000	-.0144	.0104	
		Drama	.0003	.00303	1.000	-.0096	.0103	
		Fanta	.0014	.00361	1.000	-.0105	.0132	
		Horro	-.0009	.00387	1.000	-.0136	.0118	
		Myste	-.0046	.00342	.972	-.0158	.0066	
		Roman	.0096	.00349	.203	-.0018	.0211	
		SciFi	-.0040	.00337	.991	-.0150	.0071	
		Thril	-.0052	.00343	.933	-.0165	.0060	
Spoken	Actio	Adven	-.0016	.00152	.996	-.0066	.0034	
		Comed	-.0077*	.00166	.000	-.0131	-.0022	
		Crime	-.0026	.00185	.965	-.0086	.0035	
		Drama	.0024	.00127	.774	-.0018	.0066	
		Fanta	-.0053	.00172	.088	-.0110	.0003	
		Horro	.0008	.00191	1.000	-.0055	.0070	
		Myste	.0009	.00158	1.000	-.0043	.0061	
		Roman	-.0007	.00164	1.000	-.0060	.0047	
		SciFi	.0013	.00154	1.000	-.0037	.0063	
		Thril	.0024	.00159	.945	-.0029	.0076	
		War	.0083*	.00197	.002	.0019	.0148	
	Adven	Actio	.0016	.00152	.996	-.0034	.0066	
		Comed	-.0061*	.00168	.017	-.0116	-.0006	
		Crime	-.0010	.00187	1.000	-.0071	.0052	
		Drama	.0040	.00130	.094	-.0003	.0083	
		Fanta	-.0037	.00175	.601	-.0095	.0020	
		Horro	.0024	.00193	.987	-.0040	.0087	
		Myste	.0025	.00160	.923	-.0028	.0078	
		Roman	.0009	.00166	1.000	-.0045	.0064	
		SciFi	.0029	.00156	.787	-.0022	.0080	
		Thril	.0040	.00161	.372	-.0013	.0092	
		War	.0099*	.00199	.000	.0034	.0164	
	Comed	Actio	.0077*	.00166	.000	.0022	.0131	
		Adven	.0061*	.00168	.017	.0006	.0116	
		Crime	.0051	.00198	.295	-.0014	.0116	
		Drama	.0101*	.00146	.000	.0053	.0149	
		Fanta	.0024	.00187	.983	-.0038	.0085	
		Horro	.0085*	.00205	.002	.0017	.0152	
		Myste	.0086*	.00174	.000	.0029	.0143	
		Roman	.0070*	.00179	.006	.0011	.0129	
		SciFi	.0090*	.00170	.000	.0034	.0146	
		Thril	.0100*	.00175	.000	.0043	.0158	
		War	.0160*	.00210	.000	.0091	.0229	
	Crime	Actio	.0026	.00185	.965	-.0035	.0086	
		Adven	.0010	.00187	1.000	-.0052	.0071	
		Comed	-.0051	.00198	.295	-.0116	.0014	
		Drama	.0050	.00167	.123	-.0005	.0104	
		Fanta	-.0027	.00204	.972	-.0094	.0039	
		Horro	.0033	.00220	.936	-.0039	.0106	
		Myste	.0035	.00192	.812	-.0028	.0098	
		Roman	.0019	.00197	.998	-.0046	.0083	
		SciFi	.0039	.00188	.656	-.0023	.0101	
		Thril	.0049	.00192	.306	-.0014	.0112	
		War	.0109*	.00225	.000	.0035	.0183	
	Drama	Actio	-.0024	.00127	.774	-.0066	.0018	
		Adven	-.0040	.00130	.094	-.0083	.0003	
		Comed	-.0101*	.00146	.000	-.0149	-.0053	
		Crime	-.0050	.00167	.123	-.0104	.0005	
		Fanta	-.0077*	.00154	.000	-.0128	-.0027	
		Horro	-.0016	.00175	.999	-.0074	.0041	
		Myste	-.0015	.00137	.995	-.0060	.0030	
		Roman	-.0031	.00144	.604	-.0078	.0017	
		SciFi	-.0011	.00133	1.000	-.0054	.0033	
		Thril	.0000	.00138	1.000	-.0046	.0045	
		War	.0059*	.00181	.049	.0000	.0119	
	Fanta	Actio	.0053	.00172	.088	-.0003	.0110	
		Adven	.0037	.00175	.601	-.0020	.0095	
		Comed	-.0024	.00187	.983	-.0085	.0038	
		Crime	.0027	.00204	.972	-.0039	.0094	
		Drama	.0077*	.00154	.000	.0027	.0128	
		Horro	.0061	.00210	.145	-.0008	.0130	
		Myste	.0062*	.00180	.029	.0003	.0121	
		Roman	.0046	.00185	.337	-.0014	.0107	
		SciFi	.0066*	.00177	.010	.0008	.0124	
		Thril	.0077*	.00181	.002	.0017	.0136	
		War	.0136*	.00215	.000	.0066	.0207	
	Horro	Actio	-.0008	.00191	1.000	-.0070	.0055	
		Adven	-.0024	.00193	.987	-.0087	.0040	
		Comed	-.0085*	.00205	.002	-.0152	-.0017	
		Crime	-.0033	.00220	.936	-.0106	.0039	
		Drama	.0016	.00175	.999	-.0041	.0074	
		Fanta	-.0061	.00210	.145	-.0130	.0008	
		Myste	.0001	.00198	1.000	-.0064	.0066	
		Roman	-.0014	.00203	1.000	-.0081	.0052	
		SciFi	.0005	.00195	1.000	-.0059	.0069	
		Thril	.0016	.00199	1.000	-.0049	.0081	
		War	.0076	.00230	.050	.0000	.0151	
	Myste	Actio	-.0009	.00158	1.000	-.0061	.0043	
		Adven	-.0025	.00160	.923	-.0078	.0028	
		Comed	-.0086*	.00174	.000	-.0143	-.0029	
		Crime	-.0035	.00192	.812	-.0098	.0028	
		Drama	.0015	.00137	.995	-.0030	.0060	
		Fanta	-.0062*	.00180	.029	-.0121	-.0003	
		Horro	-.0001	.00198	1.000	-.0066	.0064	
		Roman	-.0016	.00172	.999	-.0072	.0041	
		SciFi	.0004	.00162	1.000	-.0049	.0057	
		Thril	.0015	.00167	.999	-.0040	.0069	
		War	.0074*	.00203	.015	.0007	.0141	
	Roman	Actio	.0007	.00164	1.000	-.0047	.0060	
		Adven	-.0009	.00166	1.000	-.0064	.0045	
		Comed	-.0070*	.00179	.006	-.0129	-.0011	
		Crime	-.0019	.00197	.998	-.0083	.0046	
		Drama	.0031	.00144	.604	-.0017	.0078	
		Fanta	-.0046	.00185	.337	-.0107	.0014	
		Horro	.0014	.00203	1.000	-.0052	.0081	
		Myste	.0016	.00172	.999	-.0041	.0072	
		SciFi	.0020	.00168	.991	-.0035	.0075	
		Thril	.0030	.00172	.841	-.0026	.0087	
		War	.0090*	.00208	.001	.0022	.0158	
	SciFi	Actio	-.0013	.00154	1.000	-.0063	.0037	
		Adven	-.0029	.00156	.787	-.0080	.0022	
		Comed	-.0090*	.00170	.000	-.0146	-.0034	
		Crime	-.0039	.00188	.656	-.0101	.0023	
		Drama	.0011	.00133	1.000	-.0033	.0054	
		Fanta	-.0066*	.00177	.010	-.0124	-.0008	
		Horro	-.0005	.00195	1.000	-.0069	.0059	
		Myste	-.0004	.00162	1.000	-.0057	.0049	
		Roman	-.0020	.00168	.991	-.0075	.0035	
		Thril	.0011	.00163	1.000	-.0043	.0064	
		War	.0070*	.00200	.024	.0005	.0136	
	Thril	Actio	-.0024	.00159	.945	-.0076	.0029	
		Adven	-.0040	.00161	.372	-.0092	.0013	
		Comed	-.0100*	.00175	.000	-.0158	-.0043	
		Crime	-.0049	.00192	.306	-.0112	.0014	
		Drama	.0000	.00138	1.000	-.0045	.0046	
		Fanta	-.0077*	.00181	.002	-.0136	-.0017	
		Horro	-.0016	.00199	1.000	-.0081	.0049	
		Myste	-.0015	.00167	.999	-.0069	.0040	
		Roman	-.0030	.00172	.841	-.0087	.0026	
		SciFi	-.0011	.00163	1.000	-.0064	.0043	
		War	.0060	.00204	.135	-.0007	.0127	
	War	Actio	-.0083*	.00197	.002	-.0148	-.0019	
		Adven	-.0099*	.00199	.000	-.0164	-.0034	
		Comed	-.0160*	.00210	.000	-.0229	-.0091	
		Crime	-.0109*	.00225	.000	-.0183	-.0035	
		Drama	-.0059*	.00181	.049	-.0119	.0000	
		Fanta	-.0136*	.00215	.000	-.0207	-.0066	
		Horro	-.0076	.00230	.050	-.0151	.0000	
		Myste	-.0074*	.00203	.015	-.0141	-.0007	
		Roman	-.0090*	.00208	.001	-.0158	-.0022	
		SciFi	-.0070*	.00200	.024	-.0136	-.0005	
		Thril	-.0060	.00204	.135	-.0127	.0007	

Based on observed means.
 The error term is Mean Square(Error) = 6.894E-5.	
*. The mean difference is significant at the .05 level.	
