# Supplementary material for: Time-synchronic comments on video streaming website reveal core structures of audience engagement in movie viewing
Source: Front Psychol. 2023 Jan 19;13:1040755. doi: 10.3389/fpsyg.2022.1040755 (PMC9893864; doi:10.3389/fpsyg.2022.1040755)
Supplement: Supplementary file 7 [file Table_2.DOCX]

**Number of movies within each genre that fall into a specific cluster.**

|  | **Cognitive Cluster 1** | **Cognitive Cluster 2** | **Emotional Cluster 1** | **Emotional Cluster 2** |
| --- | --- | --- | --- | --- |
| **Action** | 21 | 41 | 33 | 29 |
| **Adventure** | 19 | 39 | 30 | 28 |
| **Comedy** | 19 | 23 | 14 | 28 |
| **Crime** | 19 | 11 | 14 | 16 |
| **Drama** | 83 | 54 | 54 | 83 |
| **Fantasy** | 20 | 17 | 18 | 19 |
| **Horror** | 14 | 13 | 20 | 7 |
| **Mystery** | 34 | 16 | 27 | 23 |
| **Romance** | 26 | 18 | 18 | 26 |
| **SciFi** | 29 | 26 | 30 | 25 |
| **Thriller** | 30 | 19 | 27 | 22 |
| **War** | 17 | 12 | 9 | 20 |
| **Total (**with multiple calculation of genres**)** | 331 | 289 | 326 | 294 |
| **Total (**without multiple calculation of genres**)** | 131 | 109 | 116 | 124 |

Note: Each genre has been calculated multiple times, because most movies are classified into more than one genre.
