# Supplementary material for: Time-synchronic comments on video streaming website reveal core structures of audience engagement in movie viewing
Source: Front Psychol. 2023 Jan 19;13:1040755. doi: 10.3389/fpsyg.2022.1040755 (PMC9893864; doi:10.3389/fpsyg.2022.1040755)
Supplement: Supplementary file 8 [file Table_3.DOCX]

**Names of movies that can Granger-cause audience responses cognitively and emotionally:**

| **Movies which Granger-cause cognitively** | **Movies which Granger-cause**  **emotionally** |
| --- | --- |
| *Big Fish* | *Arrival* |
| *Edge of Tomorrow* | *Flight (I)* |
| *Eternal Sunshine of the Spotless Mind* | *Captain America The First Avenger* |
| *Ex Machina* | *Cast Away* |
| *Gone Girl* | *Crazy Stupid Love* |
| *Happy Death Day* | *Edward Scissorhands* |
| *He’s Just Not That into You* | *Green Book* |
| *Justice League* | *Home Alone (I)* |
| *Kill Bill Vol.1* | *Independence Day* |
| *Knocked Up* | *I, Robot* |
| *La La Land* | *La La Land* |
| *Man Of Steel* | *Now You See Me (I)* |
| *Marriage Story* | *Oblivion (I)* |
| *Moon* | *Rear Window* |
| *Prometheus* | *Saving Private Ryan* |
| *Rise of The Planet of the Apes* | *Sleepy Hollow* |
| *The Avengers* | *Spider-Man* |
| *The Butterfly Effect* | *The Chronicles of Narnia: The Lion, The Witch, and The Wardrobe* |
| *The Curious Case of Benjamin Button* | *The Imitation Game* |
| *The Grand Budapest Hotel* | *The Pursuit of Happiness* |
| *The Hurt Locker* | *Thor* |
| *The Meg* | *The Secret Life of Walter Mitty* |
| *The Pianist* | *The Avengers.* |
| *The Prestige* |  |
| *The Purge* |  |
| *Training Day.* |  |
