# Supplementary material for: Time-synchronic comments on video streaming website reveal core structures of audience engagement in movie viewing
Source: Front Psychol. 2023 Jan 19;13:1040755. doi: 10.3389/fpsyg.2022.1040755 (PMC9893864; doi:10.3389/fpsyg.2022.1040755)
Supplement: Supplementary file 9 [file Table_4.pdf]

Examples of time-synchronic comments:

Cluster	Topic	Comment Example	Translation	Movie
128	Audience Interaction	麻烦不要开头就剧透，请有点道德。	Please be ethical. Don't spoil the movie at the beginning!	***Happy Death Day***
		这电影讲的都是什么，谁来说说?	Who can tell me what does the movie talk about?	***Blade Runner 2049***
		我觉得很多事情不需要知道后果，只要想就去做。	I think that we don't need to consider much about the consequences. If we want to do something, just do it.	***Forrest Gump***
		不要着急离开，还有彩蛋。	Don't leave in a hurry. There is a credit cookie!	***Gravity***
		你能想象吗，这是临场发挥。	Can you imagine that this is improvisation?	***Iron Man***
112	Female Characters	斯嘉丽约翰逊好漂亮。	Scarlett Johansson is so beautiful.	***Marriage Story***
		龙妈这个演员总是把她生活的表情带入角色。	Emilia Clarke always take her real life facial expressions into acting.	***Me Before You***
		这个女主角是不是演过傲慢与偏见?	Hasn't the female character starred in Pride and Prejudice?	***Love Actually***
		她居然不要武器，她是不是傻?	Is she stupid? She doesn't want a weapon!	***Prometheus***
		红皇后很好的，只是她讨厌别人对她说谎。	Red Queen is actually a nice person, but she really hates lies.	***Alice in Wonderland***
37	Movie Genre-Movie Production	温子仁的恐怖片都好看的。	James Wan's horror movies are good.	***The Dead Silence***
		这是我科幻的启蒙片。	This is my sci-fi primer.	***Contact***
		编、导、演、摄、美、录音，都是教科书级的。	Screenplay, direction, action, shooting, stage design, and recording are all perfect and classic.	***Citizen Kane***
		这不是恐怖片，这是搞笑片。	This is not a horror movie, it is a comedy.	***Shaun of the Dead***
		我觉得是最好看的科幻片没有之一。	I think it is the best scifi movie. It is uncomparable.	***Passengers***
124	Death	这人后面死的好惨。	The man will die in a painful way later.	***The Mummy***
		他如果不跑就不会死。	If he didn't run, he would not die.	***Residen tEvil Apocalypse***
		知道自己的死法后，人在更多的事情面前会更胆大。	If someone knows the way he dies, he will be braver when confronted with a lot of things.	***Big Fish***
		镇上的人全部被做成蜡像了，没一个活的。	All people in the village were made into wax figures, and no one survived.	***House of Wax***
		谁受益最大谁就是凶手呗。	Who benefits most from the murder, who is the murderer.	***Knives Out***
26	China-China's Relationship with other Countries	那个时候中国连自行车都还没有。	At that time in China, we didn't even have bicycles.	***Pearl Harbor***
		我觉得中国的恐怖片和美国恐怖片最相似的就是那音乐。	I think the most similar thing between Chinese horror movies and American horror movies is the music.	***Signs***
		二战德国在军事上从来没有和中国合作过。	Germany never cooperated with China in military during World War II.	***Dunkirk***
		从这个电影可以看出中国当年打日本有多困难。	From this movie, we can see how difficult it was for China to beat Japan back then.	***1917***
		我想中国和美国深度合作，世界进步会更加快，探索宇宙的脚步会更快。	I think if China and the United States cooperate deeply, the world will progress faster, and the pace of exploration of the universe will be faster.	***The Martian***
123	Male Charaters	羡慕男主有这种想象力。	I envy the male protagonist for having such an imagination.	***The Secret Life of Walter Mitty***
		这男的真可悲，成为坏人的工具。	This man is so pathetic. He becomes a tool for the bad guys.	***The Da Vinci Code***
		他的表情让我看到了绝望。	I see despair in his face.	***Cast Away***
		斯内普很好，他才是承受最多的人。	Snape is a good guy. He is the one who bears the most.	***Harry Potter***
		男主是不是有幻想症啊?	Does the male protagonist have delusional disorder?	***The Machinist***
132	Audience Check-in	广东人前来打卡。	Guangdong people come to check-in.	***American History X***
		有四川南充的吗?	Is there anyone from Sichuan Nanchong?	***The Day After Tomorrow***
		地址在昆明，来观看哈哈。	I'm from Kunming. I come to watch this movie, haha.	***Gone With The Wind***
		广西老乡你们好。	Fellows from Guangxi, hello.	***Black Hawk Down***
		我是江西南昌的。	I'm from Jiangxi Nanchang.	***Das Boot***
118	Parent-Child Relationship	做父亲的能理解这种父爱。	A father can understand this kind of love.	***Catch Me If You Can***
		外国人很多家里有孩子也会领养的。	Many foreigners who already have their own children will also adopt children.	***Orphan***
		既然他害怕为什么不告诉妈妈真相?	Since he was afraid, why didn't he tell his mother the truth?	***The Sixth Sense***
		根治教育问题要父母和社会大环境两手抓。	If we want to solve the problem of education, we should consider both parental and social factors.	***Elysium***
		要尊重孩子的梦想，自信的孩子会很聪明	We should respect children's dreams. Confident children will be smart.	***Charlie and The Chocolate Factory***
106	Money	希望这个是值得花钱的片子。	I hope this movie is worth the money.	***Blade Runner 2049***
		有钱可以为所欲为。	If you are rich, you can do whatever you want.	***Ready Player One***
		还是懂法律和金融的懂得生财之道啊。	It is true that people who understand law and finance can earn a lot of money.	***The Shawshank Redemption***
		钱不是万能的，但没钱往往是不能的。	Money is not all-mighty, but you can do nothing without money.	***The Pursuit of Happiness***
		大家别买，这片子很难看。	Please don't pay money for this movie. It is really bad.	***Split***

77	War-Military	战争都是恐怖的。	Wars are all terrifying.	***Inglorious Bastards***
		二战转折点是中途岛战役。	The turning point of World War II was the Battle of Midway.	***Pearl Harbor***
		看见了吗，战争时你我都是路边奔跑的平民。	Did you see that? During the war you and I would be civilians running by the roadside like them.	***Black Hawk Down***
		气氛一直压抑窒息，这就是战乱。	The atmosphere has been suppressed and suffocated, and this is war.	***Dunkirk***
		现在世界上有好多地方还是在打仗。	Now there are numbers of regions around the world which are having wars.	***Gone With The Wind***
99	Food	一个喜欢吃花生酱的死神哈哈哈哈!	He is a Death who likes peanut butter, hahahaha!	***Meet Joe Black***
		这大鱿鱼看着就好吃。	The large squid looks delicious.	***The Thing***
		梦龙不是雪糕吗?	Isn's Magnum a brand of ice-cream?	***Me Before You***
		这个意大利面看起来很好吃的样子。	The spaghetti looks really delicious.	***Hancock***
		这么像包饺子。	The food looks like dumpling.	***Maleficent***
10	Amusement	金凯瑞好逗啊哈哈哈哈。	Jim Carrey is funny, hahahaha.	***The Truman Show***
		很好笑的，不信往下看。	It is really amusing, please continue watching.	***Home Alone***
		怎么这么搞笑?	How it can be so funny?	***Forrest Gump***
		我真的看笑了哈哈哈哈!	I really want to laugh when watching this hahahaha!	***Psycho***
		美式幽默，哈哈哈哈!	American style humor, hahahaha!	***Hacksaw Ridge***
109	Marriage-Family	现在我的婚姻就是只剩下欺骗，希望接下来的人生好好的。	Now the only thing left in my marriage is cheating. I hope I can live happily in the rest of my life.	***He's Just Not That Into You***
		非常真实。要想做自己千万别结婚。	It is true. If you want to be yourself, don't get married.	***Marriage Story***
		俩人三观不一致，也不知道当初是怎么结婚的。	The two have different views. I can't understand why they get married.	***Revolutionary Road***
		男的不离婚是因为女的有钱。	The man don't want to divorce because the woman is rich.	***Gone Girl***
		说到底还是因为丈夫的不信任才会有这样的悲剧。	Anyway, it is because of her husband's distrust that such a tragedy occurs.	***Orphan***
127	Favorable Comments on Movies	看到这就想给这个作品满分，演员很棒。	When I see this I want to give full grade for this movie. The actors are really great.	***Three Billboards***
		这个要耐心看，后面真的很好看。	You have to be patient. The movie will be better later.	***Forrest Gump***
		可以，编剧想法不错。	Good. The screenwriter has good ideas.	***Tenet***
		看过很多遍还是很感动。	I have watched the movie many times, and I am still moved so much.	***Blood Diamond***
		好看好看，昆汀厉害啊!	Excellent! Quentin is really a good filmmaker!	***Kill Bill Vol. 1***
86	Fear	我已经开始害怕了。	I start to feel scared.	***The Meg***
		开头真的好诡异惊悚的感觉。	The beginning is really creepy and scary.	***Amelie***
		啊啊啊啊啊好吓人啊!	Ahhhhhhh it is really scary!	***The Ring***
		天啊太恐怖了!	Oh my god it is horrifying!	***District 9***
		一个人看有点害怕。	I am watching it alone, and I am scared.	***The Ring***
